# Deciphering difficult-to-treat psoriatic arthritis (D2T-PsA): a GRAPPA perspective from an international survey of healthcare professionals

**DOI:** 10.1093/rap/rkae074

**Published:** 2024-06-19

**Authors:** Andre L Ribeiro, Shikha Singla, Vinod Chandran, Nicholas Chronis, Wilson Liao, Christine Lindsay, Enrique R Soriano, Philip J Mease, Fabian Proft

**Affiliations:** Division of Rheumatology, University of Toronto, Women’s College Hospital, Toronto, ON, Canada; Department of Rheumatology, Medical College of Wisconsin, Milwaukee, WI, USA; Division of Rheumatology, Department of Medicine, University of Toronto, Toronto, Canada; Psoriatic Arthritis Program, Schroeder Arthritis Institute, University Health Network, Toronto, Canada; Psoriatic Arthritis Program, Schroeder Arthritis Institute, University Health Network, Toronto, ON, Canada; Department of Dermatology, University of California San Francisco, San Francisco, USA; GRAPPA Patient Research Partner, Prosper, TX, USA; Rheumatology Section, Internal Medicine Service, Hospital Italiano de Buenos Aires, Buenos Aires, Argentina; Swedish Medical Center/Providence St, Joseph Health and University of Washington School of Medicine, Seattle, USA; Department of Gastroenterology, Infectiology and Rheumatology (including Nutrition Medicine), Charité—Universitätsmedizin Berlin, Berlin, Germany

**Keywords:** spondyloarthritis, therapy, bDMARDs, csDMARDs, treatment failure, difficult to treat

## Abstract

**Objectives:**

This study contributes to the Group for Research and Assessment of Psoriasis and Psoriatic Arthritis (GRAPPA)’s effort to define ‘difficult-to-treat’ PsA (D2T-PsA), leveraging insights of healthcare professionals who are GRAPPA members. The primary objective is to inform GRAPPA’s D2T PsA project, ensuring the consensus definition reflects clinical experience and expertise.

**Methods:**

An online survey was conducted among GRAPPA’s healthcare professionals managing PsA patients. The survey covered demographic details, structured questions, and open-ended queries to gather comprehensive insights into the experts’ viewpoints.

**Results:**

About 223 physicians completed the survey, comprising 179 (80.2%) rheumatologists and 40 (17.9%) dermatologists. The majority, 184 (82.5%), favoured establishing distinct definitions for D2T-PsA and complex-to-manage PsA (C2M-PsA). Furthermore, 202 (90.5%) supported a definition that includes objective inflammation signs (clinical, laboratory, imaging, among others). However, opinions varied on the criteria for prior treatment failures, with most (93, 41.7%) favouring a definition that includes at least one conventional synthetic disease-modifying anti-rheumatic drug and two or more biological- or targeted-synthetic-DMARDs with different mechanisms of action.

**Conclusion:**

The survey reveals a majority opinion among GRAPPA experts favouring the differentiation between D2T-PsA and C2M-PsA, and the inclusion of objective inflammatory markers in these definitions. However, there is less than 50% agreement on the specific treatment failure criteria, particularly regarding the number of therapies needed to classify PsA as D2T. These findings suggest a need for continued discussion to reach a more unified approach in defining D2T-PsA, reflecting the complexity of the condition.

Key messages

- This survey found strong agreement among GRAPPA members on distinguishing between D2T- and C2M-PsA.
- Most GRAPPA members supported including objective inflammatory markers in the D2T concept to objectively differentiate it from C2M-PsA.
- Considerable variability regarding the number of treatment failures required to meet the criteria for D2T-PsA was observed, underscoring the necessity of continued research.

## Introduction

Psoriatic arthritis (PsA) is a heterogeneous autoimmune disorder, presenting with symptoms that span from the musculoskeletal system to cutaneous manifestations and involvement of other organs [1]. Significant advancements in targeted immunomodulatory therapy have been achieved in the field of PsA [2, 3]. Despite considerable progress in PsA treatments, the enduring challenge of achieving effective disease control still poses a major concern [4]. For instance, a contemporary systematic review and meta-analysis that assessed 258 publications reported a remission prevalence of only 23.1% when measured by the DAPSA score [5]. Factors such as disease activity status, comorbidities, adverse reactions to therapy and limited treatment accessibility contribute to these challenges [6, 7].

A taskforce from the EULAR has recently proposed a definition of difficult-to-treat (D2T) RA. It encompasses various criteria, including active disease, inability to taper glucocorticoid therapy, radiographic progression and/or impaired quality of life despite the use of ≥2 biological- or targeted synthetic disease-modifying antirheumatic drugs (b/tsDMARDs) with different mechanisms of action (MOA) following failure of conventional synthetic drugs (csDMARDs). Additionally, this definition also includes situations where the disease is deemed problematic by either the rheumatologist or the patient [8]. Similarly, the Assessment of SpondyloArthritis International Society (ASAS) has also launched a project that focuses on defining D2T axial spondyloarthritis (axSpA) [9].

Concurrently, the Group for Research and Assessment of Psoriasis and Psoriatic Arthritis (GRAPPA) has initiated a key project to define ‘difficult-to-treat’ psoriatic arthritis (D2T-PsA). This initiative began with a scoping review to understand existing definitions and applications of D2T-PsA in the literature [10]. An important outcome of this review was the introduction of the term ‘complex-to-manage psoriatic arthritis’ (C2M-PsA), which encompasses inclusion of factors such as depression, fatigue and comorbidities that can contribute to persistence of negative symptoms or treatment limitations. Understanding and defining D2T- and C2M-PsA is essential to address the difficulties faced by a considerable subset of patients whose conditions do not respond adequately to standard therapies. To this end, we distributed a survey to GRAPPA members to gather expert insights that will help define D2T and C2M-PsA more precisely. Additionally, an upcoming survey will capture patient perspectives to address the unique challenges and complexities these patients encounter. This dual approach, combined with the findings from the scoping literature review, aims to thoroughly inform the research group so a comprehensive definition can be developed. Such definitions will enhance identification and categorization of this patient group in clinical research, impacting clinical trial design and guideline development. By laying this groundwork, we aim to ensure that patients with resistant forms of the disease receive tailored and effective treatment recommendations. Additionally, we seek to enable regulatory agencies and pharmaceutical companies to design studies that not only involve newer drugs but also explore the potential of combining biologics and targeted synthetic disease-modifying anti-rheumatic drugs (tsDMARDs). Ultimately, this initiative intends to better address the specific needs of this patient subgroup, thereby influencing clinical trial designs and guideline development.

## Methods

The primary objective of this survey was to collect healthcare professionals’ opinions on potential criteria for defining D2T-PsA and to gain insights into their experiences in managing such cases. The study received approval from the University Health Network Research Ethics Board. Participation was limited to GRAPPA members involved in the clinical care of PsA patients and was entirely voluntary, with no financial incentives provided. Participants were given the freedom to skip any question or to withdraw from the survey at any point. To ensure confidentiality, the survey was conducted anonymously.

The development of the survey involved interactive discussions among GRAPPA members and patient research partners (PRPs), whose contributions were paramount in shaping the questions of the survey to ensure they are comprehensive and that they address the practical challenges faced by patients with PsA. These discussions informed the creation of an electronic, online questionnaire, which was hosted on the Research Electronical Data Capture (REDCap) platform and was presented in English. The survey comprised three sections: demographic information, structured questions and open-ended questions, designed to elicit comprehensive responses (see Supplementary Data S1, available at *Rheumatology Advances in Practice* online).

For the structured questions, we employed a descriptive analysis to identify common patterns and responses. In contrast, the open-ended questions were analysed thematically using Excel. This tool helped in organizing and categorizing the qualitative data, enabling the manual identification of themes and patterns for more in-depth narrative insights. This combined approach ensured that both quantitative and qualitative findings were captured, providing a well-rounded understanding of the global challenges in treating PsA.

## Results

**Figure 1.**
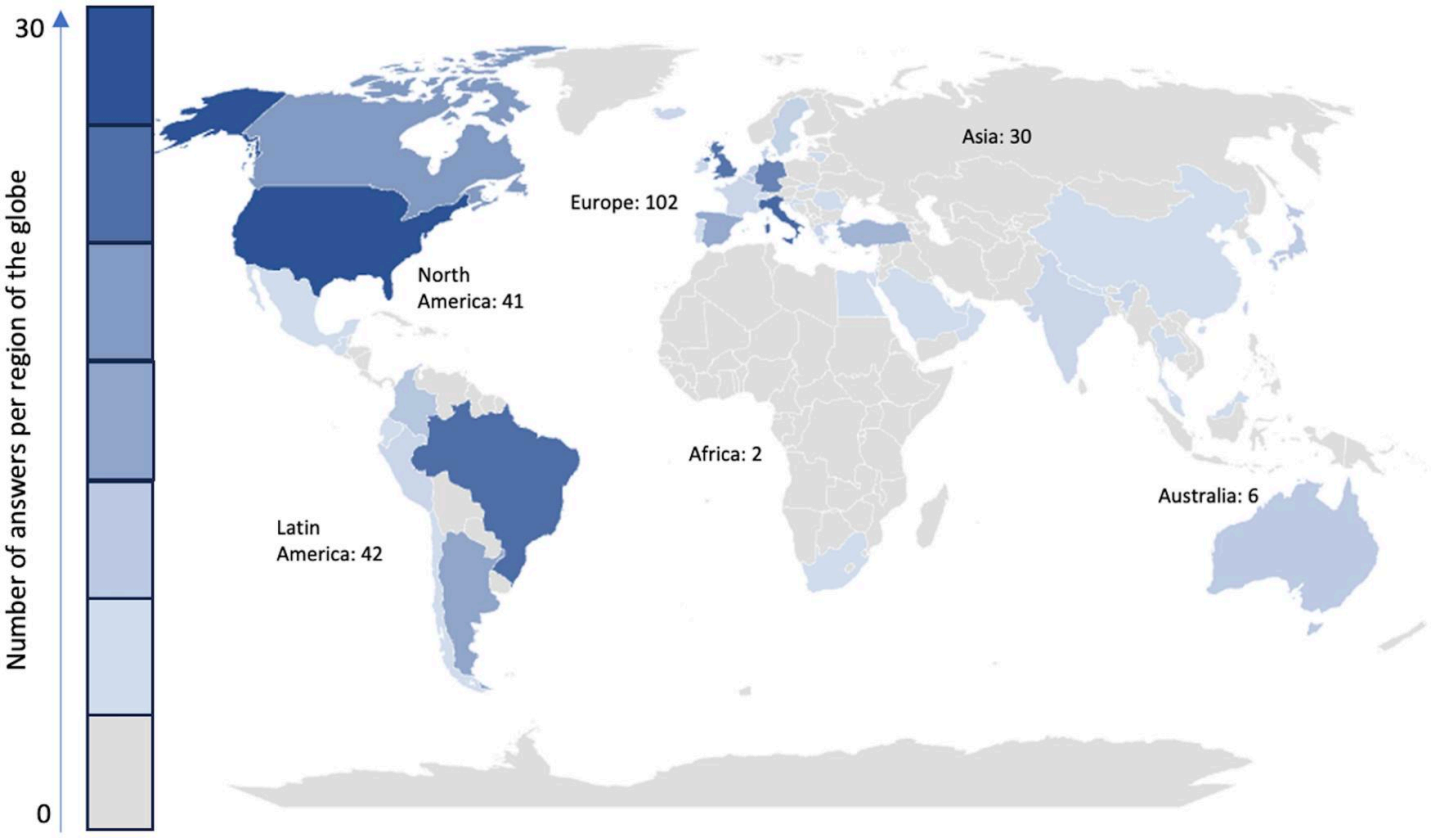
Distribution of survey respondents by region

The survey was completed by 223 GRAPPA members across 47 countries. This wide geographical spread included significant representation from most regions of the globe, including Europe (102 respondents, 45.7%), Latin America (41, 18.8%), North America (41, 18.3%), Asia (30, 13.4%), along with inputs from Africa (2, 0.9%) and Oceania (6, 2.6%) (Fig. 1). The majority of the participants were either rheumatologists (179, 80.2%) or dermatologists (40, 17.9%), complemented by four members from other related specialties (1.8%). Detailed demographic data, providing a thorough breakdown of respondent profiles, are presented in Table 1.

**Table 1.** Demographic characteristics of the GRAPPA experts answering the survey

Number of full respondents	*n* = 223
Region of respondents, *n* (%)	
Europe	102 (45.7)
North America	41 (18.3)
Latin America	42 (18.8)
Asia	30 (13.4)
Africa	2 (0.9)
Oceania	6 (2.6)
Gender, *n* (%)	
Male	142 (63.6)
Female	81 (36.3)
Age groups, *n* (%)	
<30 years	7 (3.1)
30–39 years	51 (22.8)
40–49 years	71 (31.8)
50–59years	36 (16.1)
>60 years	57 (25.5)
Medical specialty, *n* (%)	
Rheumatologists	179 (80.2)
Dermatologists	40 (17.9)
Others	4 (1.8)
GRAPPA membership, *n* (%)	
Early career member	58 (26.1%)
Full member	165 (73.9%)
Years of practice, by groups *n* (%)	
0–5 years	37 (16.5)
6–10 years	35 (15.6)
11–15 years	39 (17.4)
>15 years	112 (50.2)
Number of PsA patients treated per month, by groups *n* (%)	
0–25	72 (32.2)
26–50	84 (37.6)
51–199	46 (20.6)
101–200	14 (6.2)
>200	7 (3.1)

### Distinct definitions of D2T- and C2M-PsA

**Figure 2.**
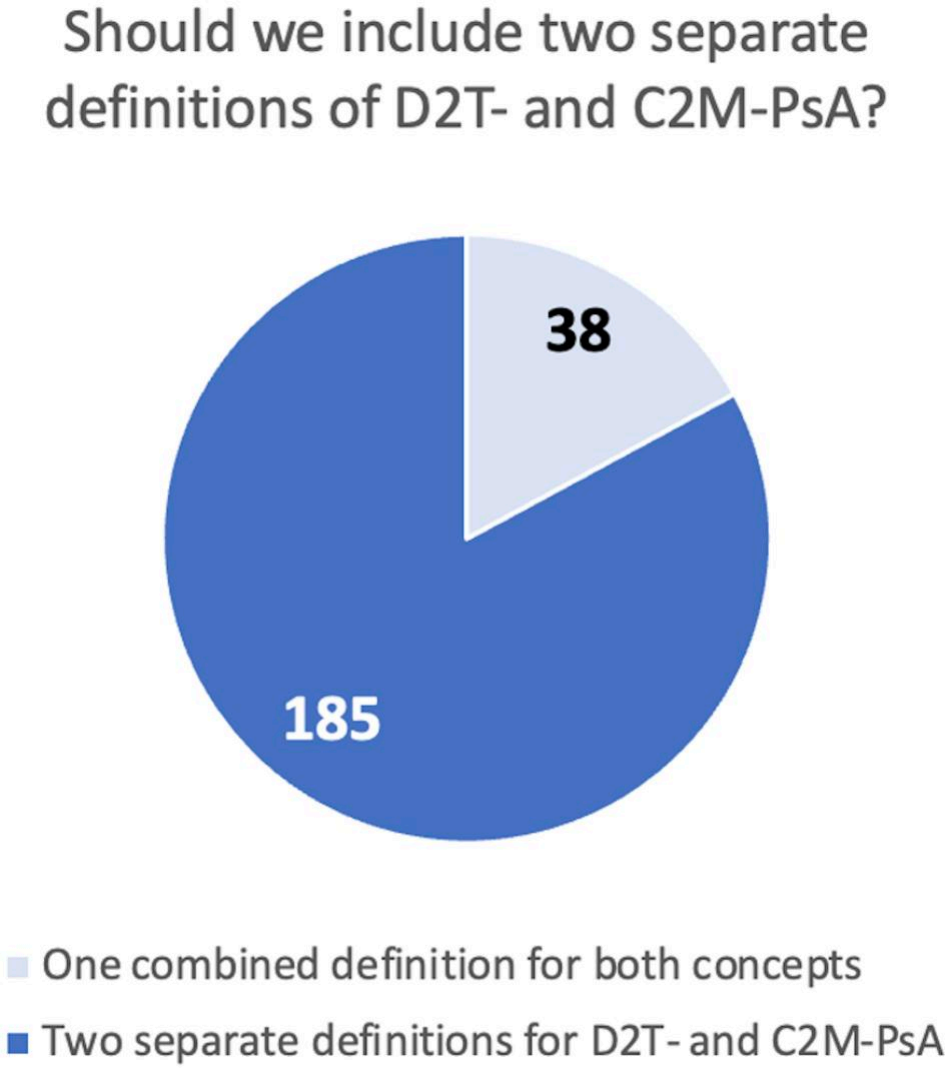
Survey responses on the distinction and definitions of D2T and C2M PsA

**Figure 3.**
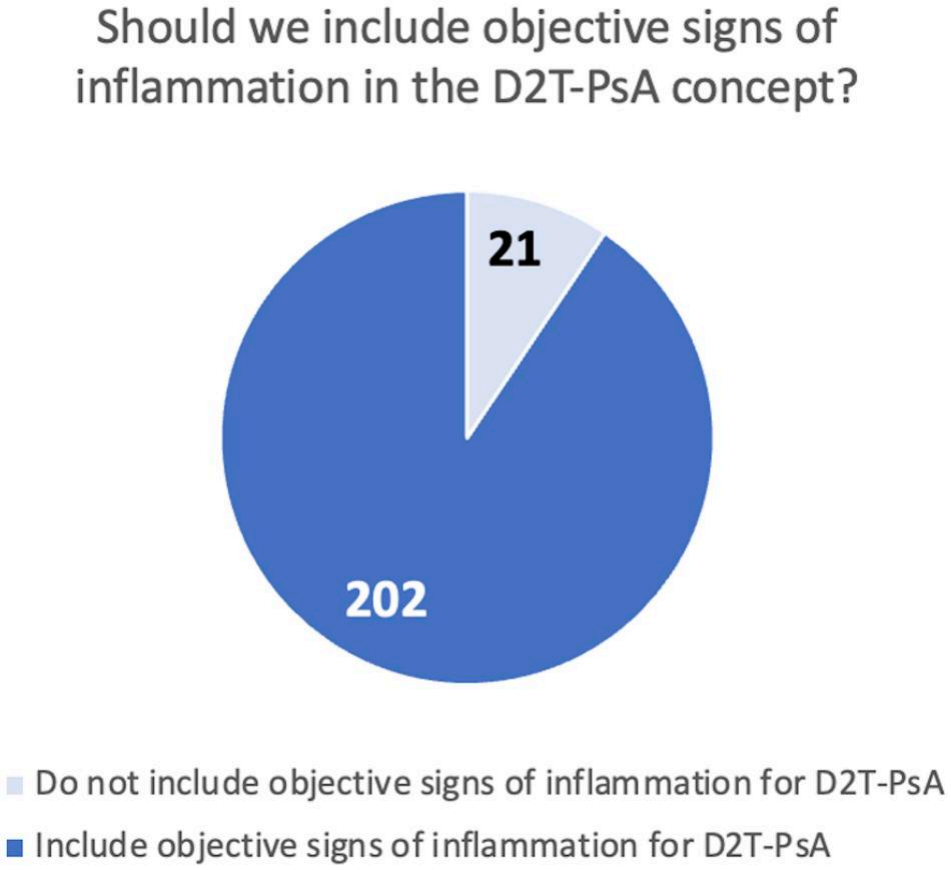
Survey responses on whether objective signs of inflammation should be a mandatory inclusion in the definition of D2T-PsA

A significant portion of respondents, 185 (82.9%), supported establishing separate definitions of D2T- and C2M-PsA (Fig. 2). Additionally, 202 (90.6%) participants recommended including objective signs of inflammation in the D2T-PsA definition (Fig. 3). Specifically, 111 (49.8%) suggested integrating persistently elevated acute phase reactants (erythrocyte sedimentation rate or C-reactive protein), and 155 (69.5%) advocated for including imaging assessments to confirm ongoing inflammation. The preferred imaging modalities are detailed in Supplementary Fig. S1, available at *Rheumatology Advances in Practice* online, with ultrasound (US, 114 answers) and magnetic resonance (MRI, 97 answers) being the most chosen methods among respondents.

### Treatments failure criteria for D2T- and C2M-PsA

**Figure 4.**
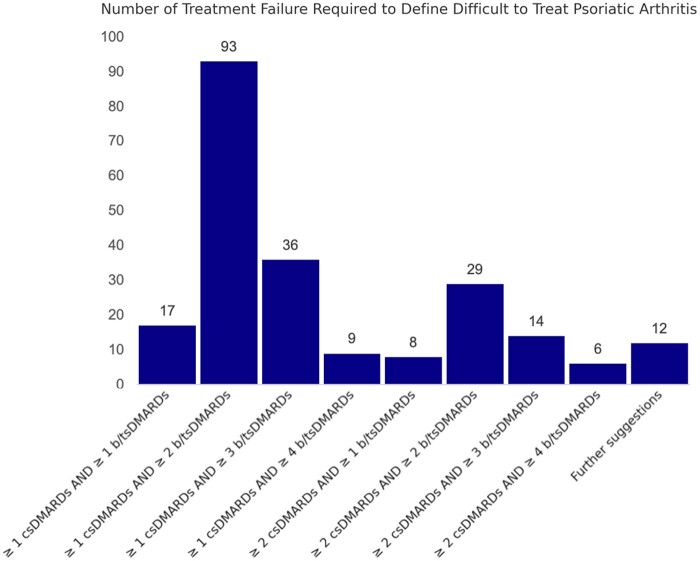
Number of treatment failures required to define a PsA case as D2T. All options highlighted are for drugs with different mechanisms of action

In defining D2T- and C2M-PsA, 149 (66.8%) of GRAPPA members emphasized that failure to at least one csDMARD should be a mandatory criterion for D2T-PsA, with 74 members (33.2%) dissenting. Furthermore, 175 (78.5%) participants preferred a definition based on failure of b/tsDMARDs with different MOAs, rather than merely counting the number of different advanced therapies used. The most favoured treatment failure criterion, chosen by 93 participants (41.7%) was the failure of ≥1 csDMARD and ≥2 b/tsDMARDs with different MOAs, while the second most common choice (36 members, 16%) involved failure of ≥1 csDMARD and ≥3 b/tsDMARDs. A detailed view of the responses concerning the number of treatment failures needed to define D2T-PsA is depicted in Fig. 4.

### Factors contributing to D2T- and C2M-PsA

**Figure 5.**
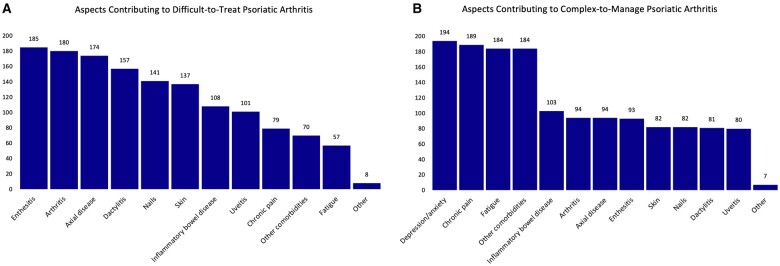
Aspects contributing to (A) D2T and (B) C2M-PsA

In assessing the factors contributing to D2T-PsA, 210 (94.2%) members identified the ineffectiveness or loss of effect of multiple medications as a major challenge, with 106 (47.5%) also emphasizing medication side effects as a prominent feature (Supplementary Fig. S2, available at *Rheumatology Advances in Practice* online). In the context of C2M-PsA, experts particularly highlighted chronic pain without evident inflammation (*n* = 167, 74.9%), disease impact on daily life (*n* = 158, 70.9%), persistent fatigue (*n* = 153, 68.6%), medication side effects (*n* = 143, 64.1%) and the inefficacy of multiple medications (*n* = 116, 52.0%) as key factors (Supplementary Fig. S3, available at *Rheumatology Advances in Practice* online). When analyzing specific disease domains, the most commonly mentioned aspects for D2T-PsA were enthesitis, arthritis, axial disease, dactylitis and skin/nail issues, followed by others. In contrast, for C2M-PsA, the ranking shifted, with depression/anxiety, chronic pain, other comorbidities and inflammatory bowel disease leading the list (Fig. 5A and B provide a detailed breakdown). The importance of drug intolerance and treatment non-adherence for defining a case as C2M was also highlighted in the survey results.

### Interdisciplinary and geographical differences

In the GRAPPA survey, rheumatologists and dermatologists displayed both congruences and divergences in their responses. Both groups showed a high level of agreement (over 80%) on the need for distinct definitions of D2T- and C2M-PsA, with a similar majority (about 90%) supporting the inclusion of objective signs of inflammation in D2T-PsA. However, notable differences emerged in their approaches. Rheumatologists, for instance, were more inclined (81.6%) to define D2T-PsA based on the variety of therapeutic mechanisms, compared with 67.5% of dermatologists. Additionally, while the majority of both groups agreed on ≥1 csDMARD + ≥2 b/tsDMARD as a criterion for D2T-PsA, a higher percentage of rheumatologists (44.1%) compared with dermatologists (32.5%) supported this definition. Moreover, rheumatologists tended to favour definitions with a higher number of failed medications for D2T classification. For example, their second most preferred definition was failing ≥1 csDMARD + ≥3 b/tsDMARDs, in contrast to dermatologists who leaned towards ≥1 csDMARD + ≥1 b/tsDMARD. The inclusion of ESR/CRP in the definition was favoured by 48.6% of rheumatologists and 57.5% of dermatologists, while the preference for including imaging was 71.5% among rheumatologists and 60% among dermatologists. The summary of responses by specialty is presented in Supplementary Table S1, available at *Rheumatology Advances in Practice* online.

In establishing two distinct definitions for PsA, most regions of the globe demonstrated significant agreement, with over 80% concordance. The incorporation of objective signs of inflammation into the definition was widely supported, exceeding 85% agreement in the included regions. Preferences for b/tsDMARDs with different MOAs over merely counting the number of drugs showed a range from 65.2% to 84.6% across these regions. The most favoured criterion for defining D2T, failing at least one csDMARD and two or more b/tsDMARDs, was favoured by 33.3–47.8% of the respondents of each included region. The next most common criterion, failing at least one csDMARD and three or more b/tsDMARDs, garnered support ranging from 4.3% in Asia to around 15.5–23% in Europe, Latin America and North America. Regarding the inclusion of drug intolerance and non-adherence in the D2T or C2M definitions, approximately 80% and 70% of respondents across the considered regions, respectively, supported this. It is important to note that this analysis excluded data from Oceania and Africa as their small sample sizes (*n* = 6 and *n* = 2, respectively) were insufficient for any meaningful sub-analysis (Supplementary Table S2, available at *Rheumatology Advances in Practice* online).

### Open-ended questions

Participants emphasized the inclusion of comorbidities like fibromyalgia, metabolic syndrome, liver disease, cardiovascular disease and malignancy in the definition of C2M. Additionally, factors such as age, gender, non-adherence and access to therapy were also deemed applicable for this definition. Non-adherence emerged as a controversial point, with some advocating its inclusion in both D2T- and C2M-PsA definitions due to its impact on treatment efficacy. Others argued for its specific relevance to C2M-PsA, attributing non-adherence to external factors like social barriers or personal beliefs rather than the disease’s complexity.

The open-ended questions also gathered insights on additional clinical issues and comorbidities relevant to PsA management. For D2T-PsA, criteria such as persistent inflammation despite b/tsDMARDs, treatment failures and specific disease activity score measures were highlighted. In contrast, for C2M-PsA, factors like chronic pain syndromes, mental health issues, socioeconomic status and healthcare access were emphasized. Some respondents also raised concerns regarding the overlap between the characteristics of D2T and C2M-PsA and brought to the fore the fact that D2T should be considered a subgroup of C2M-PsA. These responses reflect the complex interplay of clinical, psychological and social factors in PsA management, highlighting the need for comprehensive, personalized treatment approaches that go beyond mere pharmacological intervention. Finally, several respondents emphasized the importance of including a temporal perspective in these definitions and of reviewing the diagnosis of PsA to avoid attributing the diagnosis of D2T- or C2M-PsA to patients without the disease. The answers are summarized in Supplementary Table S3, available at *Rheumatology Advances in Practice* online.

## Discussion

This study addresses a significant gap in the current understanding of PsA by seeking to define D2T- and C2M-PsA. Highlighting the diverse perspectives among GRAPPA members who are experts in managing PsA patients, our findings underscore the lack of consensus on several key aspects of PsA management. The absence of universally accepted definitions for D2T- and C2M-PsA has posed a significant obstacle in clinical and research settings, leading to varied approaches in patient care and study designs. The establishment of clear criteria and definitions is essential not only for the advancement of patient care, but also to provide guidance to regulatory agencies and pave the way for novel research study designs. The results of this study are particularly relevant for informing the ongoing GRAPPA initiative to create a definition of D2T- and C2M-PsA. Furthermore, they highlight the need for continued collaborative efforts in this area.

Although both categories were agreed upon by 82.9% of respondents, the survey results, particularly responses from the open questions that are detailed in Supplementary Table S3, available at *Rheumatology Advances in Practice* online, suggest a nuanced understanding. A key theme that emerged was the indication that while D2T- and C2M-PsA are related, they are not mutually exclusive. Specifically, some experts view D2T-PsA as a subset within the broader category of C2M-PsA. This understanding underscores the concept that D2T-PsA represents a truly treatment refractory group that belongs to the wider C2M-PsA spectrum, requiring different approaches and considerations for effective management.

A notable 90.6% of participants emphasized the importance of including objective signs of inflammation in the D2T definition. Nearly half of the respondents suggested integrating ESR or CRP. This absence of agreement may stem from the fact that these markers are elevated in about half of the patients and previous studies have shown that these markers do not consistently correlate with disease activity and may not be reliable indicators of active inflammation in PsA patients [11–13]. Furthermore, approximately 70% of the participants advocated for including imaging assessments to confirm ongoing inflammation in the D2T-PsA definition. This emphasis gains relevance in light of recent studies highlighting significant discrepancies between clinical assessments and imaging findings [14]. Such discrepancies could be attributed to the presence of chronic pain and enthesalgia arising from various aetiologies, such as osteoarthritis or fibromyalgia [15]. Concerning which imaging modality to use, most respondents preferred either US or MRI. This aligns with findings from a sub-study of The Tight Control of Inflammation in Psoriatic arthritis (TICOPA) trial, which reported that both US and MRI are effective in measuring disease activity outcomes [13]. In contrast, conventional radiography and computerized tomography are limited to assessing structural damage and don’t evaluate active inflammation [16]. Finally, fluoroscopy and scintigraphy are neither widely used nor recommended in this context [16].

In evaluating the factors contributing to D2T- and C2M-PsA, the findings of this survey provide significant insights. A majority of 94% of respondents defended that ineffectiveness or loss of effect of multiple medications constitutes D2T-PsA. Specific disease domains frequently associated with D2T-PsA include enthesitis, arthritis, axial disease, dactylitis, and skin/nail issues. These align closely with the findings from a literature review by Mease and Coates, which identified five clinical domains (synovitis, enthesitis, dactylitis, spondylitis, and psoriasis/nail psoriasis) critical in evaluating remission in PsA [17]. In contrast, for C2M-PsA, the emphasis shifts to factors like depression/anxiety, chronic pain, fatigue, other comorbidities and inflammatory bowel disease, indicating a broader, more holistic view of the disease’s impact [18–20].

Medication side effects have emerged as a significant concern in both D2T- and C2M-PsA, with 47.5% of members identifying them as a key issue in D2T-PsA and 64% in C2M-PsA. This underscores the dilemma about whether to categorize medication side effects under D2T- or C2M-PsA, or both. Furthermore, the role of treatment non-adherence in defining C2M-PsA was pointed out by 44% of the respondents, highlighting its impact on disease management. The importance of non-adherence in PsA management cannot be overstated. It significantly affects remission rates and is associated with higher medical costs [21, 22]. Therefore, the survey results highlight the need for better identifying non-adherence, and also the necessity for strategies that improve patient adherence to optimize both clinical outcomes and healthcare resource utilization in PsA management.

The criteria for treatment failure in D2T-PsA also gained significant attention. Almost 70% of respondents concurred that failure to respond to at least one csDMARD should be a mandatory criterion for D2T PsA. This highlights the role of csDMARDs as a foundational element in PsA management and sets a clear benchmark for escalating treatment, being in accordance with the most recent treatment guidelines [23, 24]. In addition to this, 78% of the participants preferred a definition based on failure of b/tsDMARDs with different MOAs. This approach underscores the complexity of PsA treatment, where simply counting the number of therapies may be insufficient without considering their MOAs [25].

The preferred criterion for D2T-PsA among participants was the failure of at least one csDMARD and two or more b/tsDMARDs with different MOAs. The fact that this criterion, despite being the most popular, was endorsed by only 38% of respondents, indicates a considerable lack of consensus within the field. This is in line with the findings of a recent international survey, where 34.8% of the respondents favoured a similar definition for D2T-PsA, emphasizing the failure of at least 2 classes of b/tsDMARDs [26]. Furthermore, these two proposed criteria align with the one endorsed by EULAR for D2T-RA, which involves the failure of ≥2 b/tsDMARDs (with different MOAs), following the failure of a csDMARD [27]. Similarly, Wendling *et al.* [28] advocated for a comparable approach in delineating D2T-axSpA, proposing the failure of either ≥2 b/tsDMARDs with different MOAs or ≥3 b/tsDMARDs regardless of their MOA, as criteria. However, as recently illustrated by a scoping review carried by our research team, there is still no consensus regarding the number of drugs that a patient must fail to be classified as D2T-PsA [10].

This ongoing debate and lack of uniformity in defining D2T-PsA have been the primary motivators for GRAPPA to initiate an effort to establish unified definition for D2T- and C2M-PsA. In recent efforts to better characterize D2T-PsA, several studies have focused on this area [27, 29, 30]. Notably, a cohort study by Philippoteaux *et al.* applied the EULAR definition of D2T-RA to describe the characteristics of D2T-PsA patients. This approach provided valuable insights but also highlighted the need for PsA-specific criteria [31]. The review by Lubrano *et al.* [30] stands out in this context, as it was the only study proposing a categorization similar to GRAPPA’s approach. This review differentiated between refractory disease due to persistent inflammation, termed ‘refractory to treatment’ PsA, and non-remission attributed to pre-existing comorbidities, referred to as D2T-PsA [30]. This distinction aligns with GRAPPA’s objective of delineating the complex landscape of PsA management and underlines the necessity of nuanced definitions that encompass the diverse clinical presentations and challenges faced by PsA patients.

The delineation of PsA into D2T and C2M categories bears profound implications for patient care, clinical research and healthcare policy. Accurately characterizing D2T-PsA is crucial for evaluating advanced treatment strategies such as combined b/tsDMARD therapy. This precise classification not only enables tailored clinical approaches—choosing between modifying biological therapies or addressing comorbid conditions like fibromyalgia—but also enhances the selection criteria for clinical trials, ensuring more homogeneous study populations and a deeper understanding of treatment efficacy. The increasing prevalence of refractory disease necessitates urgent attention from regulatory agencies and pharmaceutical companies. It raises the importance of advocacy for the approval of combination therapy trials, balancing potential efficacy with safety concerns. Moreover, these distinctions assist healthcare providers, insurance companies and policymakers in making informed decisions about treatment access and funding. By providing clear treatment categorization criteria, we aim to establish evidence-based policies that appropriately weigh the benefits of advanced therapies against risks like increased infection rates, thereby optimizing clinical outcomes and resource utilization.

Our study, while providing valuable insights, does have some limitations. First, the survey was restricted to GRAPPA members, which might limit the generalizability of the findings to a broader clinical context. Second, the data relies solely on expert opinion, which, while invaluable, does not encompass patient perspectives or empirical clinical outcomes. Additionally, the survey questions were not validated due to the absence of similar validated questionnaires in the literature, potentially impacting the specificity and interpretability of the responses.

Despite these limitations, our study has several notable strengths. The survey captures the perspectives of healthcare professionals on addressing D2T-PsA, achieving higher response rates than similar surveys previously conducted by GRAPPA [32]. This suggests an increasing interest and recognition of the importance of this topic within the professional community. Significantly, the survey was distributed globally, drawing responses from diverse healthcare professionals across different regions, thereby providing a broad, international perspective on PsA management. Another major strength of the study is its interdisciplinary nature. Contributions came not only from rheumatologists, but also from dermatologists. This interdisciplinary approach enriches the survey data, offering a more comprehensive understanding of PsA. The involvement of different specialties ensures that various aspects of PsA, from musculoskeletal symptoms to skin manifestations, are adequately represented and considered in the discussion. Such a diverse range of viewpoints is crucial for developing a well-rounded and effective approach to managing PsA, famously known for being a multifaceted and heterogeneous disease.

In conclusion, this study marks a pivotal advancement in understanding and managing D2T- and C2M-PsA, underscoring the necessity for precise definitions. Despite some variations in opinions, our global and interdisciplinary survey, engaging both rheumatologists and dermatologists, revealed significant agreements, notably on differentiating between D2T- and C2M-PsA and including objective inflammation markers in D2T definitions. However, consensus was not reached regarding the number of treatment failures necessary to meet the D2T-PsA definition. Furthermore, incorporating patient perspectives is essential to ensure that research outcomes are relevant and beneficial to those directly affected. In this project, PRPs are playing an integral role, not only in the design and implementation of the questionnaires but also in every stage of the research process. Going forward, these collaborations will continue to play a pivotal role as we continue with the next step of the project, namely an international patient survey aiming to capture the perspective of multiple patients from various regions around the globe. Together with the scoping literature review and the here presented GRAPPA HCP survey, these results shall facilitate the following Delphi process within the GRAPPA community, which will help establish a unified, consensus-driven definitions for D2T and C2M-PsA, thereby advancing treatment strategies and clinical research in the field of PsA.

## Supplementary Material

rkae074_Supplementary_Data

## Data Availability

All data relevant to the study are included in the article or uploaded as supplementary information.

## References

[1] Kishimoto M , Deshpande GA, Fukuoka K et al Clinical features of psoriatic arthritis. Best Pract Res Clin Rheumatol 2021;35:101670. 33744078 10.1016/j.berh.2021.101670

[2] Ortolan A , Lorenzin M, Cozzi G et al Treat-to-target in real-life psoriatic arthritis patients: achieving minimal disease activity with bDMARDs/tsDMARDs and potential barriers. Semin Arthritis Rheum 2023;62:152237. 37453183 10.1016/j.semarthrit.2023.152237

[3] Ayan G , Ribeiro A, Macit B, Proft F. Pharmacologic treatment strategies in psoriatic arthritis. Clin Ther 2023;45:826–40. 37455227 10.1016/j.clinthera.2023.05.010

[4] Zardin-Moraes M , da Silva ALFA, Saldanha C et al Prevalence of psoriatic arthritis patients achieving minimal disease activity in real-world studies and randomized clinical trials: systematic review with metaanalysis. J Rheumatol 2020;47:839–46. 31575702 10.3899/jrheum.190677

[5] Hagège B , Tan E, Gayraud M et al Remission and low disease activity in psoriatic arthritis publications: a systematic literature review with meta-analysis. Rheumatology (Oxford) 2020;59:1818–25. 32118267 10.1093/rheumatology/keaa030

[6] Ribeiro AL , Dullius L, Sartori NS et al Challenges in the management of psoriatic arthritis in Latin America: a systematic review. Clin Ther 2023;45:860–7. 37198042 10.1016/j.clinthera.2023.04.005

[7] Ng BCK , Jadon DR. Unmet needs in psoriatic arthritis. Best Pract Res Clin Rheumatol 2021;35:101693. 34099367 10.1016/j.berh.2021.101693

[8] Nagy G , Roodenrijs NM, Welsing PM et al EULAR definition of difficult-to-treat rheumatoid arthritis. Ann Rheum Dis 2021;80:31–5. 33004335 10.1136/annrheumdis-2020-217344 PMC7788062

[9] ASAS definition of difficult-to-treat axial spondyloarthritis. https://www.asas-group.org/asas-definition-of-difficult-to-treat-axial-spondyloarthritis/ (24 December 2023, date last accessed).

[10] Singla S , Ribeiro A, Torgutalp M et al Difficult-to-treat psoriatic arthritis (D2T-PsA): a scoping literature review informing a GRAPPA research project. RMD Open 2024;10:e003809. 38191215 10.1136/rmdopen-2023-003809 PMC10806599

[11] Punzi L , Podswiadek M, Oliviero F et al Laboratory findings in psoriatic arthritis. Reumatismo 2007;59:52–5. 17828345 10.4081/reumatismo.2007.1s.52

[12] Houttekiet C , de Vlam K, Neerinckx B, Lories R. Systematic review of the use of CRP in clinical trials for psoriatic arthritis: a concern for clinical practice? RMD Open 2022;8:e001756. 35135860 10.1136/rmdopen-2021-001756 PMC8830278

[13] Helliwell PS , Coates LC, Chew NS et al Comparing psoriatic arthritis low-field magnetic resonance imaging, ultrasound, and clinical outcomes: data from the TICOPA trial. J Rheumatol 2020;47:1338–43. 31676693 10.3899/jrheum.181385

[14] Sarabia S , Farrer C, Yeung J et al The pattern of musculoskeletal complaints in patients with suspected psoriatic arthritis and their correlation with physical examination and ultrasound. J Rheumatol 2021;48:214–21. 32414953 10.3899/jrheum.190857

[15] Macchioni P , Salvarani C, Possemato N et al Ultrasonographic and clinical assessment of peripheral enthesitis in patients with psoriatic arthritis, psoriasis, and fibromyalgia syndrome: the ULISSE study. J Rheumatol 2019;46:904–11. 30877205 10.3899/jrheum.171411

[16] Mandl P , Navarro-Compán V, Terslev L et al; European League Against Rheumatism (EULAR). EULAR recommendations for the use of imaging in the diagnosis and management of spondyloarthritis in clinical practice. Ann Rheum Dis 2015;74:1327–39. 25837448 10.1136/annrheumdis-2014-206971

[17] Mease PJ , Coates LC. Considerations for the definition of remission criteria in psoriatic arthritis. Semin Arthritis Rheum 2018;47:786–96. 29566966 10.1016/j.semarthrit.2017.10.021

[18] Magrey MN , Antonelli M, James N, Khan MA. High frequency of fibromyalgia in patients with psoriatic arthritis: a pilot study. Arthritis 2013;2013:762921. 23476767 10.1155/2013/762921 PMC3586452

[19] McDonough E , Ayearst R, Eder L et al Depression and anxiety in psoriatic disease: prevalence and associated factors. J Rheumatol 2014 May;41:887–96. 24692521 10.3899/jrheum.130797

[20] Campanholo CB , Maharaj AB, Corp N et al Management of psoriatic arthritis in patients with comorbidities: an updated literature review informing the 2021 GRAPPA treatment recommendations. J Rheumatol 2023;50:426–32. 36319003 10.3899/jrheum.220310

[21] Ferreira MF , Kohem CL, Xavier RM et al Treating psoriatic arthritis to target: discordance between physicians and patients’ assessment, non-adherence, and restricted access to drugs precluded therapy escalation in a real-world cohort. Clin Rheumatol 2019;38:961–8. 30511296 10.1007/s10067-018-4383-9

[22] Pasma A , Schenk C, Timman R et al Does non-adherence to DMARDs influence hospital-related healthcare costs for early arthritis in the first year of treatment? PLoS One 2017;12:e0171070. 28152001 10.1371/journal.pone.0171070 PMC5289489

[23] Coates LC , Soriano ER, Corp N et al; GRAPPA Treatment Recommendations Domain Subcommittees. Group for Research and Assessment of Psoriasis and Psoriatic Arthritis (GRAPPA): updated treatment recommendations for psoriatic arthritis 2021. Nat Rev Rheumatol 2022;18:465–79. 35761070 10.1038/s41584-022-00798-0 PMC9244095

[24] Gossec L, Baraliakos X, Kerschbaumer A et al EULAR recommendations for the management of psoriatic arthritis with pharmacological therapies: 2019 update. Ann Rheum Dis 2020;79:700–12. 10.1136/annrheumdis-2020-217159 PMC7286048 32434812

[25] Merola JF , Lockshin B, Mody EA. Switching biologics in the treatment of psoriatic arthritis. Semin Arthritis Rheum 2017;47:29–37. 28363434 10.1016/j.semarthrit.2017.02.001

[26] Marzo-Ortega H , Harrison SR, Nagy G et al Time to address the challenge of difficult to treat psoriatic arthritis: results from an international survey. Ann Rheum Dis 2024;83:403–4. 37963707 10.1136/ard-2023-225087

[27] Perrotta FM , Scriffignano S, Ciccia F, Lubrano E. Clinical characteristics of potential “difficult-to-treat” patients with psoriatic arthritis: a retrospective analysis of a longitudinal cohort. Rheumatol Ther 2022;9:1193–201. 35612694 10.1007/s40744-022-00461-w PMC9314519

[28] Wendling D , Verhoeven F, Prati C. Is the difficult-to-treat (D2T) concept applicable to axial spondyloarthritis? Joint Bone Spine 2023;90:105512. 36528335 10.1016/j.jbspin.2022.105512

[29] Kumthekar A , Ashrafi M, Deodhar A. Difficult to treat psoriatic arthritis—how should we manage? Clin Rheumatol 2023;42:2529–30. 37097525 10.1007/s10067-023-06605-9

[30] Lubrano E , Scriffignano S, Perrotta FM. Difficult to treat and refractory to treatment in psoriatic arthritis. Rheumatol Ther 2023;10:1119–25. 37395952 10.1007/s40744-023-00574-w PMC10468455

[31] Philippoteaux C , Marty-Ane A, Cailliau E et al Characteristics of difficult-to-treat psoriatic arthritis: a comparative analysis. Semin Arthritis Rheum 2023;63:152275. 37852155 10.1016/j.semarthrit.2023.152275

[32] Song K , Webb L, Eder L et al Screening/referral strategies for the early recognition of psoriatic arthritis (PsA) among patients with psoriasis: results of a GRAPPA survey. J Rheumatol 2023;50:1439–45. 37582554 10.3899/jrheum.2023-0424

