# Supplementary material for: Deciphering difficult-to-treat psoriatic arthritis (D2T-PsA): a GRAPPA perspective from an international survey of healthcare professionals
Source: Rheumatol Adv Pract. 2024 Jun 19;8(3):rkae074. doi: 10.1093/rap/rkae074 (PMC11193309; doi:10.1093/rap/rkae074)
Supplement: rkae074_Supplementary_Data [file rkae074_supplementary_data.docx]

**Supplementary material**

**Index**

**Supplementary Data S1.** [**Healthcare practitioners survey.** 2](#_Toc164834772)

[**Supplementary Table S1. Answers by specialty.** 6](#_Toc164834773)

[**Supplementary Table S2. Answers by region of the globe.** 7](#_Toc164834774)

[**Supplementary Table S3. Open-ended questions and answers.** 9](#_Toc164834775)

[**Supplementary Figures** 27](#_Toc164834776)

# **Supplementary Data S1. Healthcare practitioners survey.**

Q1 - Please indicate the gender you identify yourself with: 1 Female; 2 Male; 3 Other.

Q2 – Please indicate the age group you belong to: 1 < 30 years; 2 30-39 years; 3 40-49 years; 4 50-59 years; 5 > 60 years

Q3 – In which country do you practice?

Q4 – What is your medical specialty? R Rheumatology; D Dermatology; O Other.

Q5 – Which type of GRAPPA membership do you hold? 1 Full membership; 2 Early Career Membership.

Q6 – How many years have you been practicing as a rheumatologist or dermatologist? 1 0-5 years; 2 6-10 years; 3 11-15 years; 4 > 15 years.

Q7 – Are you a trainee or consultant/attending? 1 Trainee; 2 Consultant/attending.

Q8 – What is your primary clinic setting? AC Academic Center; CC Community Center; PC Private Clinic; C Combined.

Q9 – How many PsA patient do you treat in a month? 1 0-25; 2 26-50; 3 51-100; 4 101-200; 5 > 200.

Q10 - From your viewpoint, would you advocate for the establishment of two distinct definitions: one for Difficult-to-Treat Psoriatic Arthritis (D2T-PsA), characterizing a state of genuine resistance to various available therapies, coupled with persistent objective signs of inflammation; and on the other hand: Di13cult-to-Manage Psoriatic Arthritis (D2M- PsA), which encapsulates a wider concept encompassing not only treatment resistance, but also elements like treatment side effects, co- existing health conditions, and other factors such as fatigue and central sensitization. 1 Yes, I support this approach of two separate definitions; 2 No, I would proceed with ONLY ONE definition taking both above mentioned concepts into account.

Perspective_q10 - Please provide us with your perspective on this.

Q11 - In your opinion, what makes psoriatic arthritis “difficult to treat”? q11___1 Ineffectiveness or loss of effect of multiple medications; q11___2 Side effects from medications; q11___3 Persistent fatigue despite control of other symptoms; q11___4 Chronic pain in the absence of inflammatory activity; q11___5 High impact of disease on daily life (work, social, etc.); q11___6 other.

Other_q11 - Please elaborate.

Q12 – In your opinion, what makes psoriatic arthritis “difficult to manage”? q12___1 Ineffectiveness of multiple medications; q12___2 Side effects from medications; q12___3 Persistent fatigue despite control of other symptoms; q12___4 Chronic pain in the absence of inflammatory activity; q12___5 High impact of disease on daily life (work, social, etc.); q12___6 Other.

Other_q12 - Please elaborate.

Q13 – Which of the following aspects contribute to “difficult to treat (D2T) PsA”? q13___1 Arthritis; q13___2 Enthesitis; q13___3 Dactylitis; q13___4 Skin; q13___5 Nails; q13___6 Axial disease; q13___7 Uveitis; q13___8 Inflammatory bowel disease (ulcerative colitis / Crohn’s disease); q13___9 Depression/anxiety; q13___10 Chronic pain; q13___11 Fatigue; q13___12 Other comorbidities (e.g.: obesity, hypertension, osteoporosis, diabetes etc.; q13___13 Other.

Other_q13 - Please elaborate.

Q14 – Which of the following aspects contribute to “difficult to manage (D2M) PsA”? q14___1 Arthritis; q14___2 Enthesitis; q14___3 Dactylitis; q14___4 Skin; q13___5 Nails; q14___6 Axial disease; q14___7 Uveitis; q14___8 Inflammatory bowel disease (ulcerative colitis / Crohn’s disease); q14___9 Depression/anxiety; q14___10 Chronic pain; q14___11 Fatigue; q14___12 Other comorbidities (e.g.: obesity, hypertension, osteoporosis, diabetes etc.; q14___13 Other.

Other_q14 - Please elaborate.

Q15 – Should the definition of difficult-to-treat (D2T) PsA include evidence of objective signs of inflammation (clinical, laboratory, imaging, or others)? 1 Yes; 0 No.

Perspective_q15 - Please provide us with your perspective on this.

Q16 – Would you include failure (intolerance + ineffectiveness) to at least one conventional synthetic DMARD (csDMARD as mandatory for the definition of difficult to treat PsA? 1 Yes; 2 No.

Q17 – When considering bDMARD/tsDMARDs failure for the definition of D2T-PsA, would you simply count the number of different advanced therapies used or would you rather sort by mode of action and support a definition based on different modes of action prescribed? 1 I would prefer a definition of D2T-PsA based simply on the number of advanced therapies used; 2 I would support a definition of D2T-PsA also based on the number of different modes of action prescribed.

Q18 – Which and how many disease-modifying antirheumatic (DMARDs) should at least be tried with insufficient response (failed) to define difficult to treat PsA: 1: ≥ 1 csDMARDs AND ≥ 1 b/tsDMARDs; 2: ≥ 1 csDMARDs AND ≥ 2 b/tsDMARDs with different modes of action; 3: ≥ 1 csDMARDs AND ≥ 3 b/tsDMARDs with different modes of action; 4: ≥ 1 csDMARDs AND ≥ 4 b/tsDMARDs with different modes of action; 5: ≥ 2 csDMARDs and ≥ 1 b/tsDMARDs; 6: ≥ 2 csDMARDs AND ≥ 2 b/tsDMARDs with different modes of action; 7: ≥ 2 csDMARDs AND ≥ 3 b/tsDMARDs with different modes of action; 8: ≥ 2 csDMARDs AND ≥ 4 b/tsDMARDs with different modes of action; 9: Further suggestions.

Suggestions_q18- Please provide us with your suggestions on this.

Q19 – Should persistent elevation of acute phase reactant (ESR or CRP) be included in the definition of difficult to treat PsA? 1 Yes; 0 No.

Q20 – Should the definition of difficult to treat PsA include imaging assessments? 1 Yes; 0 No.

Q21 – If yes, which imaging modality should be included in the definition (select all that apply)? Q21___1 Ultrasound; q21___2 MRI; q21___3 X ray; q21___4 CT; q21___5 Any of the above; q21___6 Other.

Other_q21- Please provide us with your perspective on this.

Q22 – Would you include drug intolerance also in the definition of difficult to treat PsA? 1 Yes; 2 No; 3 Include in the definition of difficult to manage only.

Elaborate_q22- Please provide us with your perspective on this.

Q23 – Would you include non-adherence also in the definition of difficult to treat PsA? 1 Yes; 2 No; 3 Include in the definition of difficult to manage only.

Elaborate_q23- Please provide us with your perspective on this.

Open_q24 – What additional clinical issues or comorbidities should be addressed in the workup and management of D2T and D2M PsA?

Open_q25 – Please define any additional characteristics and suggest criteria for D2T PsA.

Open_q26 - Please define any additional characteristics and suggest criteria for D2M PsA.

# **Supplementary Table S1. Answers by specialty.**

| Rheumatologists – 179 | Dermatologists – 40 |
| --- | --- |
| Q10 (Establishment of Two Distinct Definitions):   - Yes: 149 responses – Yes Percentage: 83.71% - No: 29 responses – No Percentage: 16.2% | Q10 (Establishment of Two Distinct Definitions):   - Yes: 33 responses – Yes Percentage: 82.5% - No: 7 responses – No Percentage: 17.5% |
| Q11 (What makes PsA D2T):   - Ineffectiveness or loss of effect of multiple medications: 172 (96%) - Side effects from medications: 88 (49.1%) - Persistent fatigue despite control of symptoms: 29 (16.2%) - Chronic pain in the absence of inflammation: 57 (31.8%) - High impact of disease on daily life: 68 (37.9%) | Q11 (What makes PsA D2T):   - Ineffectiveness or loss of effect of multiple medications: 35 (87.5%) - Side effects from medications: 18 (45%) - Persistent fatigue despite control of symptoms: 11 (12.5%) - Chronic pain in the absence of inflammation: 17 (42.5%) - High impact of disease on daily life: 26 (65%) |
| Q12 (What makes PsA C2M):   - Ineffectiveness or loss of effect of multiple medications: 93 (51.9%) - Side effects from medications: 116 (64.8%) - Persistent fatigue despite control of symptoms: 126 (70.3%) - Chronic pain in the absence of inflammation: 135 (75.4%) - High impact of disease on daily life: 129 (72%) | Q12 (What makes PsA C2M):   - Ineffectiveness or loss of effect of multiple medications: 21 (52.5%) - Side effects from medications: 23 (57.5%) - Persistent fatigue despite control of symptoms: 25 (62.5%) - Chronic pain in the absence of inflammation: 30 (75%) - High impact of disease on daily life: 28 (70%) |
| Q13 (What makes PsA D2T):   - Arthritis 147 (82.1%) - Enthesitis 150 (83.7%) - Dactylitis 130 (72.6%) - Skin 115 (62.2%) - Nails 109 (60.8%) - Axial disease 137 (76.5) - Uveitis 89 (49.7%) - Inflammatory bowel disease 94 (52.5%) - Depression/anxiety 43 (24%) - Chronic pain 55 (30.7%) - Fatigue 37 (20.6%) - Other comorbidities 56 (31.2%) | Q13 (What makes PsA D2T):   - Arthritis 29 (72.5%) - Enthesitis 32 (80%) - Dactylitis 24 60%) - Skin 18 (45%) - Nails 29 (72.5%) - Axial disease 34 (85%) - Uveitis 11 (27.5%) - Inflammatory bowel disease 12 (30%) - Depression/anxiety 13 (32.5%) - Chronic pain 22 (55%) - Fatigue 15 (37.5%) - Other comorbidities 13 (32.5%) |
| Q13 (What makes PsA C2M):   - Arthritis 75 (41.8%) - Enthesitis 73 (40.7%) - Dactylitis 66 (36.8%) - Skin 69 (38.5%) - Nails 63 (35.1%) - Axial disease 75 (41.8%) - Uveitis 61 (34%) - Inflammatory bowel disease 79 (44.1%) - Depression/anxiety 156 (87.1%) - Chronic pain 156 (87.1%) - Fatigue 151 (84.3%) - Other comorbidities 147 (82.1%) | Q13 (What makes PsA C2M):   - Arthritis 18 (45%) - Enthesitis 19 (47.5%) - Dactylitis 15 (37.5%) - Skin 12 (30%) - Nails 18 (45%) - Axial disease 19 (47.5%) - Uveitis 18 (45%) - Inflammatory bowel disease 22 (55%) - Depression/anxiety 34 (85%) - Chronic pain 29 (72.5%) - Fatigue 29 (72.5%) - Other comorbidities 28 (70%) |
| Q15 (Definition to Include Objective Signs of Inflammation):   - Yes: 162 (90.5%) - No: 17 (9.5%) | Q15 (Definition to Include Objective Signs of Inflammation):  - Yes: 36 (90.0%)  - No: 4 (10.0%) |
| Q16 (Inclusion of csDMARD):   - Yes: 119 (66.5%) - No: 60 (33.5%) | Q16 (Inclusion of csDMARD):   - Yes: 29 (72.5%) - No: 10 (25%) |
| Q17 (Count therapies or MOA):   - Number of therapies: 33 (18.4%) - Therapies with d/ MOA: 146 (81.6%) | Q17 (Count therapies or MOA):   - Number of therapies: 12 (30%) - Therapies with d/ MOA: 27 (67.5%) |
| Q18 (Number of therapies required):   - ≥ 1 csDMARD + ≥ 1 b/tsDMARD: 9 (5%) - ≥ 1 csDMARD + ≥ 2 b/tsDMARD: 79 (44.1%) - ≥ 1 csDMARD + ≥ 3 b/tsDMARD: 32 (17.8%) - ≥ 2 csDMARD + ≥ 2 b/tsDMARD: 24 (13.4%) | Q18 (Number of therapies required):   - ≥ 1 csDMARD + ≥ 1 b/tsDMARD: 7 (17.5%) - ≥ 1 csDMARD + ≥ 2 b/tsDMARD: 13 (32.5%) - ≥ 1 csDMARD + ≥ 3 b/tsDMARD: 4 (10%) - ≥ 2 csDMARD + ≥ 2 b/tsDMARD: 4 (10%) |
| Q19 (Inclusion of ESR and/or CRP):   - Yes: 87 (48.6%) - No: 92 (51.4%) | Q19 (Inclusion of ESR and/or CRP):   - Yes: 23 (57.5%) - No: 16 (40%) |
| Q20 (Inclusion of imaging):   - Yes: 128 (71.5%) - No: 51 (28.5%) | Q20 (Inclusion of imaging):   - Yes: 24 (60%) - No: 15 (37.5%) |
| Q21 (Imaging modality):   - Ultrasound: 97 (54.1%) - MRI: 81 (45.2%) - X-ray: 19 (10.6%) - CT: 4 (2.2%) - Any of the above or other: 25 (13.9%) | Q21 (Imaging modality):   - Ultrasound: 14 (35%) - MRI: 13 (32.5%) - X-ray: 9 (22.5%) - CT: 3 (7.5%) - Any of the above or other: 11 (27.5%) |
| Q22 (Inclusion of drug intolerance):   - Yes: 79 (44.1%) - No: 26 (14.5%) - Only in the C2M definition: 74 (41.4%) | Q22 (Inclusion of drug intolerance):   - Yes: 16 (40%) - No: 8 (20%) - Only in the C2M definition: 15 (37.5%) |
| Q23 (Inclusion of non-adherence):   - Yes: 36 (20.1%) - No: 64 (35.8%) - Only in the C2M definition: 79 (44.1%) | Q23 (Inclusion of non-adherence):   - Yes: 7 (17.5%) - No: 17 (42.5%) - Only in the C2M definition: 15 (37.5%) |

# **Supplementary Table S2. Answers by region of the globe.**

| Q10 (Establishment of Two Distinct Definitions): Yes Percentage   - Africa 100% - Asia 87% - Oceania 57.1% - Europe 83.5% - North America: 82.1% - Latin America: 81.8% |
| --- |
| Q15 (Definition to Include Objective Signs of Inflammation): Yes Percentage   - Africa 100% - Asia 87% - Oceania 71.4% - Europe 89% - North America: 94.9% - Latin America: 95.5% |
| Q16 (Inclusion of csDMARD): Yes Percentage   - Africa 100% - Asia 65.2% - Oceania 71.4% - Europe 61.5% - North America: 69.2% - Latin America: 75% |
| Q17 (Count therapies or MOA):  Count therapies: Count mechanism of action:   - Africa 0% 100% - Asia 34.7% 65.2% - Oceania 16.5% 82.5% - Europe 27.2% 72.7% - North America 15.3% 84.6% - Latin America. 28.5% 71.4% |
| Q18 (Number of therapies required): Africa Asia Europe Latin Am. North Am. Oceania   - ≥ 1 csDMARD + ≥ 1 b/tsDMARD: 0% 8.6% 5.5% 11.3% 7.6% 0% - ≥ 1 csDMARD + ≥ 2 b/tsDMARD: 0% 47.8% 43.1% 38.6% 33.3% 71.4% - ≥ 1 csDMARD + ≥ 3 b/tsDMARD: 0% 4.3% 15.5% 20.4% 23.0% 0% - ≥ 2 csDMARD + ≥ 2 b/tsDMARD: 100% 8.6% 15.5% 13.6% 5.12% 14.2% |
| Q19 (Inclusion of ESR and/or CRP): Yes Percentage   - Africa 0% - Asia 56.5% - Oceania 14.3% - Europe 45.9% - North America: 43.6% - Latin America: 68.2% |
| Q20 (Inclusion of imaging): Yes Percentage   - Africa 100% - Asia 69.6% - Oceania 57.1% - Europe 67.9% - North America: 71.8% - Latin America: 72.7% |
| Q22 (Inclusion of drug intolerance):  Country Yes Perc. No Perc. Only in C2M   - Africa 100% 0% 0% - Asia 47.8% 13.0% 39.1% - Europe 44.0% 13.7% 41.2% - Latin Am. 40.9% 18.1% 40.0% - North Am. 41.0% 12.8% 46.1% - Oceania 28.5% 42.8% 28.5% |
| Q23 (Inclusion of non-adherence):  Country Yes Perc. No Perc. Only in C2M   - Africa 0% 100% 0% - Asia 21.7% 39.1% 39.1% - Europe 15.5% 33.0% 50.4% - Latin Am. 29.5% 36.3% 34.0% - North Am. 17.9% 35.8% 46.1% - Oceania 14.2% 85.7% 0% |

# **Supplementary Table S3. Open-ended questions and answers.**

| From your viewpoint, would you advocate for the establishment of two distinct definitions? Please elaborate. | - The management include all the armamentarium available for the treatment, therapy included.  - Biologically difficult-to-treat PsA is a subgroup of D2M and should be derived from this group using a standardised approach, which is not defined yet. So the correct way would be to define D2M and to develop (as a next stem) the guidance how to come from D2M to D2N.  - The narrower definition will allow for more robust studies with defined endpoints.  - Difficult-to-treat can be included in the concept of difficult-to-manage patients as treatment resistance patient profile, but I guess it's more common we deal with difficult to manage patient than with just treatment resistance.  - I think a wider concept has to involve all the characteristics described in difficult to manage patients, that include the concept of Difficult to treat patients, and simplify the clinical concepts.  - D2T-PSA is the non-responder and is a separate consideration to responders who get AEs.  - Not to produce too much titles to remember.  - The 2 are distinct but in reality, it is more of a continuum.  - What is the objective of such a proposal. In my view it is to identify patients with refractory disease and objective inflammation who need new treatment options. We. cannot possibly identify, manage, and treat all the myriad of conditions that influence symptomatology. So, what is the point of the second, additional, definition?  - I prefer the wider term as it addresses issues that are very important for patients more than the (lack of enough) therapeutic alternatives.  - D2T PsA is my preferred definition, encompasses more objective disease related features as determined by physician, D2M includes more subjective features fatigue, and treatment side effects can be subjective as described by patient.  - In my opinion, D2T-PsA and D2M-PsA are closely related concepts and it would be difficult to differentiate between them in clinical practice. In view of many overlapping features, would vote to keep one definition only.  - D2T PsA.  - In my view the majority of patients with PsA are difficult to manage due to the heterogenous nature of the disease and high prevalence of comorbidities.  - I prefer to establish only the concept of "difficult - to- treat (D2T) PSA". Difficult-to-manage patients would have such a wide spectrum of problems, requiring diverse individualized approach, which make it difficult to establish a single definition / concept.  - I'm doubting about these two definitions. Because are central sensitization and fatigue not also signs of disease activity? Objective signs of inflammation like swelling (combined with pain) of the joints, are not only signs of disease activity. More over tender or painful joints without swelling, painful enthesis/tendons, fatigue are sign of disease activity. Why differentiation between objective signs of inflammation and other signs of disease activity?  - I think having two separate definitions are states in which there is a huge degree of overlap unnecessarily complicates things.  - Keep it simple.  - Let's not complicate an issue.  - The management is more difficult than treat, by the way. Further anti-IL, everything became more simple and best handling.  - I prefer Difficult-to-Manage Psoriatic Arthritis (D2M-PsA), which encapsulates a wider concept.  - I think that one definition will be preferable.  - We should keep it simple.  - I think these two terms are very overlapping. With our treatment we want to manage the disease.  - I would proceed with only one definition because there is some overlap between them and the second is wider and includes more information.  - It'll be difficult for almost all people outside of GRAPPA to know how they differ and which is which.  - Make it simple.  - I would use difficult to manage PsA.  - Both approaches are hard to treat.  - Splitting the concept of d2t in two based on the presence of "true" refractoriness (however defined) will result in one clearly defined group of patients (d2t) as opposed to a very heterogeneous one (d2m), where patients who are failing therapies for a plethora of reasons might be classified into. It would make much more sense to keep a single "difficult to treat" category, to which one or more of a limited number of labels can be applied to describe the reasons of refractoriness (e.g., Chronic pain condition, comorbidities that sustain inflammation, etc.). Only then would we have the means to select subgroups of d2t patients that could benefit from different interventions and test them separately on those.  - To avoid confusion and because of considerable overlap.  - I think two definitions are not necessary.  - I believe the definition of "genuine" treatment resistance is very interpretation-prone and borderline impossible, given the fact that the number of biomarkers confirming "real" inflammatory activity is limited (clinical examination is unreliable, CRP is frequently normal even in "active" patients, and imaging such as US and particularly MRI requires additional expertise and might be not accessible (particularly MRI). Moreover, studies show that other factors apart from inflammatory activity might play more significant role for PSA patients as compared with other conditions such as RA and AS.  - "Treat" and "Manage" translation in Portuguese have practically the same meaning, and would probably take time to explain. In my opinion, important concepts should be easily understood.  - Many a times these both situations overlap. It may not be possible in clinical practice to discern between persistent inflammation and element of central sensitization. We therefore need to have a single definition.  - Likely both concepts will be contributing to a significant subset of patients. We can look signs to classify as D2T, but D2M will be the exclusion of D2T. |
| --- | --- |
| In your opinion, what makes psoriatic arthritis “difficult to treat”? Please elaborate. | - Heterogeneous nature of disease attempting to establish its extent.  - Comorbidities.  - Unable to use Biologics due to cost factor.  - Presence of joint damage related to PsA.  - Progression of structural damage despite therapy (also if therapy is clinically effective).  - Side effects/Intolerance or Contra-indications need to be well defined and should be rather restrictive.  - Comorbidities that impact treatment choice.  - Need studies to collect data for MOA.  - Absence of relevant biomarkers for patients’ stratification.  - Presence of comorbidities (e g. obesity, diabetes) or other external factors sustaining inflammation (e.g., smoke).  - Low compliance; low accessibility of medical care / treatments.  - I am concerned by the concept of "objective inflammation" without definition in this survey. ESR and CRP are fairly frequently normal with objective inflammation. |
| In your opinion, what makes psoriatic arthritis “complex to manage”? Please elaborate. | - Heterogeneous disease with multiple approaches.  - Health care access restrictions.  - Co-morbidities.  - Non-compliance.  - Comorbidities.  - Comorbidities preventing/inhibiting treatment (e.g., infections/cancer/obesity); also, non-adherence.  - Comorbidities and psychosocial factors.  - Other comorbidities that limit choice of drugs or cause failure of therapies.  - Unable to use of Biologics due to cost factor as well as no insurance coverage.  - Comorbidities.  - Comorbidities.  - Comorbidity that contraindicates effective treatment.  - Probably all items make it difficult to manage, but then again, I would not call a PsA patient with chronic pain due to fibromyalgia a "D2M PsA", but rather a "D2M Fibromyalgia".  - Co-morbidities (e.g., obesity, osteoarthrosis, degenerative spine disease).  - Limited access to therapies in specific scenarios.  - Unknown physio pathological relationship between comorbidities and PsA.  - Coexisting comorbidity and risk stratification related to PsO and PsA.  - Refractoriness and disability related to associated conditions; Disability or relevant clinical importance related to comorbidities (obesity, depression, metabolic syndrome).  - Patient reluctance to start appropriate therapy due to fear of side effects, multiple comorbidities.  - Ongoing problems with obesity.  - Reduced access to healthcare services, rheumatologists, medications.  - Co-morbidities that limit treatment options.  - Poor compliance, Co-morbidities limiting treatment options, unrealistic expectations, co-existing mental health issues affecting pharmacological therapy and compliance.  - Low compliance; low accessibility of medical care / treatments.  - Comorbidities.  - Chronic bursal inflammation (e.g., trochanteric bursae).  - High impact of social factors on disease activity and therapy adherence.  - Comorbidities as your original definition proposed.  - NAFLD, who's often present as a part of metabolic syndrome, makes csDMARDs problematic. |
| Which of the following aspects contribute to “difficult to treat (D2T) PsA”? Please elaborate. | - Comorbidities - depends on comorbidity - if prevents use of certain treatments then this contributes.  - Osteoarthrosis, obesity (causing strain on joints, pain and inactivity).  - Again, absence of relevant biomarker for treatment choices in a precision medicine approach.  - Severe disease with radiographic damage.  - Refractory biologic.  - Osteoarthritis, crystal arthropathy.  - Social factors.  - While skin is general well controlled with current therapies, difficult to treat PsA patients, skin may also be challenging. |
| Which of the following aspects contribute to “difficult to manage (C2M) PsA”? Please elaborate. | - Non-compliance.  - Non-compliance.  - Economic and social issues; adverse effects of drugs including infections.  - All comorbidities that result in limited treatment options.  - Osteoarthrosis, obesity (causing strain on joints, pain and inactivity).  - Social factors.  - Soft tissue inflammation (e.g., chronic bursitis). |
| Should the definition of difficult-to-treat (D2T) PsA include evidence of objective signs of inflammation (clinical, laboratory, imaging, or others)? Please elaborate. | - ESR and/or CRP are often normal.  - Best to have objective evidence of treatment non-response e.g., ultrasound or MR scan. Blood biomarkers are not particularly informative.  - Clinical inflammation or ultrasonography/MRI demonstrating enthesitis and/or synovitis  - Radiologic progression despite optimized treatment, elevated CRP and VHS despite treatment, doppler activity despite treatment and clinical signs of arthritis despite treatment, primary failure to more than one biologic  - Obj signs of inflammation can be difficult to come by Also the diagnosis of PsA is very difficult for a dermatologist. In elderly patients we often see a mix of arthrosis and PsA.  - Clinically reproducible, tender and swollen joints and objective signs of enthesitis and dactylitis.  - It important to determine if the source of symptoms is coming from the "psoriatic inflammation" rather than anatomical/radiographic changes. It is crucial to determine if treatment should be based only or mostly on synthetic/bio DMARDS or a pain relieving / modifying approach should be taken.  - Laboratory measures obviously unrealisable but important to include. Imaging could have an asterisk to mention US and MRI findings that would support activity.  - Disease activity must be objectively present.  - Not all symptoms are due to active PsA - although some manifestations e.g., enthesitis are more difficult to 'prove'  - All clinical, laboratory or imaging data that is directly related to psoriatic disease or extracutaneous or extra-articular manifestations  - US, MRI, CRP elevated.  - Important to differentiate between inflammation and other causes of symptoms. However always tricky to have "objective" evidence as clinical exam may vary.  - Veg - pcr imágenes con sacroilitis o cambios de entesitis o sinovitis por ecografia evidencia de signos de enfermedad activa por reumatologo o dermatologo.  - Arthritis, dactylitis, enthesitis, uveitis, fatigue and skin affection.  - Important to have something that you can objectively measure.  - If there is going to be a distinction between difficult to treat and difficult to manage, then the distinction will be blurred in the absence of objective signs of inflammation -- e.g. a patient with chronic back pain, sacroiliitis and enthesitis on exam, etc.  - Serum cytokine levels might be helpful. CRP and ESR are usually not so much helpful in the population I have.  - For it to be really considered difficult-to-treat, after multiple bDMARDs with different mechanisms of actions, I would require imaging evidence of persistent inflammation (ultrasound for peripheral and MRI for axial).  - Physical examination, composite scores.  - To help to distinguish between chronic nociplastic pain and inflammatory pain.  - High inflammatory markers and or presence of Doppler positive USG.  - DAPSA BASDAI MASES BASMI PASI BSA.  - Artritis, entenmsitis, dactilitis, VSG/PCR elevadas.  - US o MRI for enthesis, MRI for axial pain, objective synovitis in clinical exam or image (US or MRI) in Joint pain or dactylitis  - Non-responder with inflammation separate issue to non-responder with nothing to find and CRP.  - CRP, ultrasound  - Means imaging showing inflammation on joint, enthesis, and clinical evidence in the skin  - Important data  - Yes, but not exclusively.  - Objective signs are for several of the domains not an option. objective is a disputable thing in this setting.  - Synovitis on US.  - To distinguish from chronic pain.  - One needs to be certain that the difficulty to treat arises from persistent disease activity rather than from other mechanisms. If a patient continues to have fatigue, despite absence of objective evidence of inflammation there may be a sleep disturbance, they may not get enough sleep, they may be anemic, they may be depressed.  - Power Doppler on Ultrasound, Raised CRP serum levels, etc.  - Should include ANY of the signs of inflammation.  - Symptoms + some measure of inflammation (mainly clinical, also acute phase & imaging). NB imaging/acute phase in absence of troublesome symptoms should NOT be included in definition (i.e., starting point is patient).  - Objective signs of inflammation are important and should be part of the definition. However, in some patients there are other issues as fibromyalgia, mood disorder without evidence of inflammation. Hence, I suggest to divide the definition to objective inflammation and to other components w/o inflammation.  - Inflammation markers are not always elevated in PsA. Also, clear definitions for imaging findings are lacking or are vague.  - Assuming the main goal of these definitions is to facilitate clinical research, objective measurements are important, but indeed only feasible if the D2T is regarded as only a subset of D2M.  - Inflammatory markers; 2. Imaging evidence of inflammation (ultrasound, MRI); 3. Synovitis or enthesitis clinically  - Active arthritis, enthesitis, dactylitis, SpA, active skin lesions.  - Pain, ESR/CRP, blood flow signal of peripheral joint ultrasound, edema of MRI.  - Without objective inflammation the persistent symptoms could be from another source.  - Disease activity and CRP and axial, sacroiliitis.  - Physician designated joint inflammation is sufficient but shoulder also specify joint damage related synovitis.  - Swelling and pain limitation, hyperpigmentation over joint tendon  - At least one objective sign of inflammation should be included to avoid the inclusion of patients with secondary chronic pain syndrome/fibromyalgia.  - High CRP, presence of swollen joints, acute dactylitis, enthesitis confirmed by US presence of sacroiliitis/spondylitis, BSA >5.  - US evidence of inflammation or high ESR/CRP should be a part of D2T definition otherwise it would be difficult to assess response in follow up visit and also objective evidence make definition clinically relevant in practice as well  - Differentiating difficult-to-treat from difficult-to-manage patients through objective signs of inflammation is particularly important in therapeutic terms. While the former could benefit from the development of new DMARD drugs aimed at anti-inflammatory action, the latter benefit more from other therapies such as physical therapy, cognitive-behavioral therapy and non-DMARD medications.  - In my point of view patients with objective signs of inflammation are in higher risk of destructive disease with loss of function in short time. A disease without inflammatory signs can end in loss of function but are less aggressive.  - Joint swelling, CRP elevation, ultrasound and MRI findings.  - Physical examination and/or Ultrasound studies.  - If proceeding with two definitions, D2T AND D2M I think they should also be clearly distinguishable from each other, and there should be a possibility to be BOTH D2T and D2M  - Clinical signs, if there are objective signs, are useful. But in a great percentage of patients, there are no evidence of objective signs. Absence of inflammation in serology does not mean that there isn't inflammation. You don't want to see erosions on imaging. And mostly PsA patients are painful but without swelling in evaluation of disease activity.  - IMAGING ULTRASOUND WITH ENTHESITIS, DATTILITIS ARTHRITIS.  - Inflammation would be the primary driver of D2T.  - I think it is more useful to have a definition that is based on objective measures if possible  - CRP is not perfect but should be in - Ultrasound proof that a painful enthesis is indeed an enthesitis and not Fibromyalgia (the same is also true for difficult to assess peripheral joints like shoulders, hips, ankles, mid foot, ...) - Imaging confirmation that back pain (extremely prevalent) is indeed true inflammatory spinal involvement (probably extremely rare).  - Objective signs of inflammation should distinguish between pain and inflammation.  - Yes, probably but I do think the issue of pain from established arthritis (but not central sensitization/ depression etc.) contributes to difficult to treat (not just difficult to manage).  - Patients with PsA often have joint pain without significant objective findings, and we have to acknowledge that, and in some patients’ joint pain without objective signs disappears with active treatment.  - Not always easy to know if there is inflammation or not  - Many difficult to treat patients have negative inflammatory markers/lab tests and using these in definitions could result in insurance/coverage issues for medications.  - Damage, US/PD detected inflammation.  - Ask patient. Photo rash and -itis  - Lab findings might be unreliable, ultrasound should not be added in such criteria, a high number of swollen joints might indicate a D2T - PsA but also a single one refractory swollen joint too, so swollen joint count might be unreliable  - Multiple joint inflammation and constitutional signs and symptoms.  - Important to separate damage from ongoing inflammation.  - Acute reactants alteration; unequivocal inflammatory imaging (enthesitis or synovitis or tenosynovitis on the US; BME or osteitis or synovitis/enthesitis/tenosynovitis on the MRI); clinical evident synovitis /enthesitis/dactylitis or unequivocal inflammatory rhythm + prolonged morning stiffness report by patient.  - If you are differentiating the 2, I think D2T would be "treatment resistant". I would include some objective findings with this such as disease activity scores, continued active domains etc. rather than just fatigue and chronic pain.  - There needs to be a distinguishing difference between D2T and D2M and by including objective signs of inflammation, perhaps D2T would be easier to be defined.  - Persistent swelling over peripheral joints. Laboratory evidence of inflammation need not be positive US or MRI evidence of inflammation at the peripheral joints and/or entheses.  - CRP serum level, but other markers of subclinical inflammation will come up (transcriptome in blood).  - We need signs of inflammation; otherwise, it's psoriasis with concomitant fibromyalgia.  - This would preclude instances where people have been on long term steroids have grumbling disease activity which will not be captured.  - Persistently raised CRP US signs of inflammation.  - At least 2 swollen joints.  - Elevation of CRP or ESR, inflammation in US or MRI.  - Observable inflammation (swollen joints, enthesitis, psoriasis) or inflammatory markers.  - Please include, but should not be mandatory to be defined as D2T. Is good to encourage clinicians to get/repeat MRI, US, labs for objective evidence. But sometimes patients don't have and still respond to advanced therapies.  - The persistence of inflammation is an objective sign that the treatment is not effective enough.  - Particularly elevated CRP values.  - Tender, swollen, CRP, +/- MRI or ultrasound detecting inflammation.  - Clinical: synovitis, tender dactylitis, persistent enthesitis, inflammatory back symptoms. If in doubt, enthesitis on ultrasound, or imaging evidence of spine inflammation. High ESR/CRP may be helpful, but should not be mandatory (given that only 20% of patients with objective signs of inflammation may have elevated ESR/CRP).  - We need objective signs of inflammation in order to discriminate between D2T from D2M.  - Clinical evidence of inflammation (joints, enthesis...). I would not consider inflammatory biomarkers (poor sensitivity, poor specificity in PsA). I would consider imaging signs of inflammation and especially US (joints, enthesis) and MRI (spine, SI).  - This emphasizes the unmet needs of effectiveness of current available therapies to achieve targets of disease control. To me, this constitutes genuine difficulties in treatment.  - Objective signs of inflammation should include US study.  - Objective signs of inflammation, clinical or through imaging, help to differentiate those true non responders to meds from those with other issues such as superimposed FMS.  - Should include evidence of objective signs of inflammation in at least one of these settings: clinical, laboratory or imaging.  - Clinical, laboratory, imaging.  - Evidence of enthesitis through ultrasound if not detectable through physical exam. evidence of arthritis through ultrasound if not detectable through physical exam. Elevated inflammation markers.  - Clinical or imaging inflammation.  - Signs and or symptoms including imaging suggestive of inflammatory activity in one or more domains.  - Pain, swelling, stiffness; clinical findings or imaging features of inflammation with or without elevated inflammatory markers.  - To encompass certain homogeneity in the patient population in regards to study populations.  - D2T is not ONLY inflammation-based, but also other aspects.  - There should be clinical and imaging evidence of active inflammation in the joints, spine or entheses.  - The number of biomarkers confirming "real" inflammatory activity is limited (clinical examination is unreliable, CRP is frequently normal even in "active" patients, and imaging such as US and particularly MRI requires additional expertise and might be not accessible (particularly MRI). Moreover, studies show that other factors apart from inflammatory activity might play more significant role for PSA patients as compared with other conditions such as RA and AS.  - Acute phase reactants, imaging, clinical inflammatory symptoms.  - Elevated inflammatory markers (such as CRP) and objective signs of inflammation in the physical examination and imaging methods.  - MRI, ultrasound.  - I think that the target of the treatment is to reduce or completely revert inflammation which is leading to disease manifestations. Instead, I see management as a strategy to improve the overall health-relates quality of life of patients living with PSA by alleviating symptoms related to the condition but not necessarily driven by active inflammation such as functional limitations due to structural damage. This may also include treatment of comorbidities and extra articular manifestations. I hope this makes sense.  - CRP, ESR, physical examination.  - Synovitis.  - Joint swelling and tenderness and erythema, dactylitis, ultrasound evidence (doppler).  - The definition of difficult to treat should include objective evidence of inflammation, however, it should not be the only criterion. If in the mind of the patient and the provider the unacceptable symptoms are secondary to the disease process without any objective evidence, that should also be counted.  - Any signs of inflammation should be considered.  - Not sure. We don't yet have the tools for this.  - Should have clearer indisputable signs of inflammation, swelling, u/s faith cpd enhancement. |
| Which and how many disease-modifying antirheumatic (DMARDs) should at least be tried with insufficient response (failed) to define difficult to treat PsA? Please elaborate. | - ≥2 b/tsDMARDs with different modes of action.  - Some patients will have comorbidities that preclude the use of csDMARD, e.g., NASH. If a patient had to fail one csDMARD to be classified as difficult to treat, then none of these patients could ever be classified as such. Thus, I would suggest 0 or 1 csDMARDs and at least three biologics comprising at least two MOAs.  - csDMARD should not be mandatory. Failure of 2 or more advanced therapies.  - ≥1 csDMARDs AND ≥3 b/tsDMARDs with at least 2 different modes of action.  - ≥1 csDMARDs AND ≥3 b/tsDMARDs.  - >= 2 b/tsDMARDs with different modes of action.  - More than 3b/ts, would not include csDMARD in definition.  - >=2 b/tsDMARDs with different mode of action.  - Would favor >or= to 2 b/tsDMARDs without the requirement for csDMARD given treatment guidelines suggesting first line use of bDMARDS.  - Mostly a suggestion for the next round... I would you a points allocation system for this question to really see how far away your responses are. It was hard to me to pick one choice... you may want to have that granularity to make a decision.  - If csDMARD is included in the description, we will miss out the patients with axial PsA only.  - As new treatments are developed DMARDs may no longer be a relevant reference or initial step in treatment. |
| Would you include drug intolerance also in the definition of difficult to treat PsA? Elaborate. | - Intolerance is defined as patient inability to tolerate a drug whether subjectively or objectively through clear lab abnormalities or clearly attributable side effects.  - D2T is a subset of C2M but these terms overlap, so perhaps D2T and C2M should be combined into one entity. If it is D2T then it is C2M. If it is C2M then it is D2T.  - Drug intolerance contributes to making PsA difficult to treat.  - The definition needs to be wide enough.  - Intolerance to multiple csDMARDs and/or b/tsDMARDs.  - Adverse events including gastrointestinal.  - Including drug intolerance seems logical, otherwise patients who are not tolerating multiple treatments could never fulfill the definition, if it would be based on number of ineffective treatments.  - Only genuine intolerance should be considered.  - I would prefer if it was included only in C2M.  - Drug intolerance many times would lead to withdrawal of therapy and not necessarily ineffective therapy. If we consider two different approaches of D2T and C2M, I would include intolerance in C2M.  - Side effects experienced such as depression or greater than 20% weight loss with apremilast. Similar side effect with other meds.  - Difficult to treat should be looking at those patients who truly do not respond to the biologic suppression of disease, not those who are intolerant of meds for other reasons (which is C2M).  - Drug intolerance is an important consideration in determining treatment's effectiveness. It is prohibitive in reaching treatment targets if a particular drug's dose cannot be optimized due to intolerance, hence this can be considered a failure of that drug /serve as a barrier to count towards treatment response.  - ESR or CRP should be included but not mandatory as some patients do not have an increase in them (even in acute disease).  - Be included in the assessment of D2T, but may not necessarily need to show inflammation to qualify as D2T.  - Drug intolerance does not reflect the disease severity it rather be considered as an idiosyncratic response hence difficult to treat related more the immune related pathways and resistance to response which can depend on the genetic, late diagnosis and etiopathogenesis etc.  - Multiple advanced therapeutic failures.  - Only true intolerance makes disease D2T.  - It could highlight a recurring concern but it fits in more with management issues than resistant disease.  - Unable to assume the drug regularly.  - This is more related to non-PsA parameters.  - Drug intolerance could go either way. If it is a minor side effect such as fatigue, hair loss, patient preference I would think of that as more of a difficult to manage issue as some of these are not even true side effects. Overall biologics are very well tolerated and it is usually more of a safety concern on the part of the patient that makes them want to stop citing these minor issues. Major side effects such as serious infections, IBD etc., play into an actual difficult to treat patient.  - Intolerable gastrointestinal symptoms; hepatotoxicity or nephrotoxicity or medullar toxicity confirmed by exams.  - Intolerance removes the ability to use the drug therapeutically (to treat).  - Only should consider major side effects.  - Regard it is same as a failure to treatment.  - Poor tolerance in the IL-17 group is challenging with only two options.  - An important aspect of treating patients with a life-long disease.  - Many drug intolerances are objective (not necessarily subjective).  - I think safety and intolerance is not an efficacy issue but a management issue.  - I think it should be included, but there should be clear definitions. A mild oral candidiasis that can be successfully treated locally should not be ticked off as intolerance to IL17i but should just be treated.  - Multiple drug intolerances in the example of allergic reactions can make PsA D2T, non-effectiveness is just as serious as effectiveness plus moderately serious side effects in my opinion.  - Drug intolerance refers to an inability to tolerate the adverse effects of a medication, generally at therapeutic or subtherapeutic doses. These adverse effects are, frequently, gastrointestinal, haematologic, or skin events.  - GI intolerance, hepatic enzymes elevation, recurrent headaches, leukopenia, thrombocytopenia, anemia.  - Drug side effects should be part of complex to manage.  - Reduces the number of possible treatments.  - Should take into account while counting the number of drugs used. Intolerance not = non-response.  - Assessing psoriatic liver (which is different in my opinion with drug - MTX tolerance), and inability to prescribe conventional.  - Drug intolerance at times is idiosyncratic. As per my opinion, these patients respond to change in medication or some alternative methods to counter intolerance.  - Side effect, patient could not take po, when used SC fever and obvious skin rash, systemic side effect, prone to infection when used  - Can be subjective.  - Not the same as adverse drug reactions, intolerance should be the problem caused by the individual specificity. For example, sb. cannot keep in applying injection to treat PsA for afraid pain.  - Compliance often an issue, patient’s claim lack of effect or toxicity, yet have no record in pharmacy of picking up drug  - D2T should be reflective of biologically refractory disease despite optimal treatment, therefore drug intolerance should not be included  - Difficult to manage should include intolerance to drugs as NAFLD for example.  - Drug intolerance should be used in difficult to manage, as it might work on the disease but the patient cannot tolerate it.  - Any side effects... G.I... Systemic, symptoms fever, idiosyncratic arthralgias, infections, thromboembolic phenomenon.  - Intolerance does not make a patient difficult to treat, as he/she may respond well and for a long time to a subsequent treatment, therefore not being considered a failure.  - Drug intolerance more associated with those with difficult to manage disease but comorbidities that are part of the SpA cohort may play a part so important to also include those in the difficult to treat group too.  - To subjective this is rather difficult to manage from my perspective.  - Many factors involved in intolerance - anxiety, depression as well as true intolerance.  - Intolerance or adverse effects to drugs should be considered difficulties in treatment, because treatment options are reduced.  - Drug intolerance may be "subjective" e.g., headaches or fatigue. But there can be specific issues like demyelination, paradoxical psoriasis, allergy which can significantly limit treatment options  - All sorts of reasons for drug intolerance. I would keep the definition focused.  - If a patient responded briskly to medications but has side effects with all of them, the patient was fairly easy to treat...  - We should stop using the concept "difficult-to-treat" and use treatment resistance or failure to be more specific for patients with this profile. Because to think about the next step we need to discriminate if there was failure or intolerance to the previous treatments and the studies should evaluate these conditions separately.  - Allergy or side effects like GIT intolerance.  - Side-effects.  it is important BUT it is not failure of inflammatory control. I would include it in difficult to manage.  complicates the issue  - Genetic basis may predispose such a condition.  - For me difficult to treat should be those patients with inflammation despite talking adequately the prescribed medication, if there is intolerance to a drug, that patient might have responded, but is unable to take it, that is difficult to manage, but is not an intrinsically resistant patient. Is different  - Real or perceived, it is important.  - Yes, but not exclusively.  - Drug intolerance is a subjective thing in most of the times  - A florid rash is diff to treat, indigestion/malaise =diff to manage  - There often are reasons why patients have drug intolerance which are not related to the therapeutic effect of the drug.  - Drug intolerance fits into difficult to manage.  - If the patient has intolerance to at least 2 drugs with different mechanism, could be considered difficult to treat.  - D2T should be restricted to true resistance to modes of action tried. |
| Would you include non-adherence also in the definition of difficult to treat PsA? Elaborate. | - Non adherence may require non-pharmacological interventions.  - Uncertainty on the appropriate administration.  - Yes, non-adherence is different from intolerance and ineffective treatment.  - I would prefer it was included in C2M, as the non-adhesion patients in my experience differs very much from difficult-to-treat patients.  - We have to take it in count, but it is not concern related with a disease that could be hard to treat. It is a patient or social environment issue.  - Compliance not linked to side effects is purely individual issue. Clinicians then have to explore the reasons and address them to improve compliance. This may lead to good response to the already using/used medication making it not D2T condition.  - Taking a medication intermittently and not regularly.  - My thoughts: D2T should encompass more objective indicators. Non-adherence is too vague and can be due to many other factors (e.g., social barriers such as costs) that are not scientific-based; hence, my preference is to include this in the C2M definition rather than D2T.  - Adverse effect, developing infection after using biologics  - It is a component of the considerations, as it precludes what might have otherwise been an effective treatment.  - Non adherence is essentially a management issue but why it should be included as a consideration in difficult to treat is because treatment gaps were identified in our study as being contributory to eventual loss of response to biologic therapy.  - Non-adherence is a multitasking problem.  - Patient behaviour.  - Drug intolerance could be due to many factors. More of a management problem than treatment issue.  - Drug intolerance would likely to result in patients discontinuing treatment, either by the clinician or by patient themselves. Similarly, clinicians would stop any ineffective drug before switching to another treatment. Essentially, the next choice of drug treatment would require more thought and consideration, which makes it more difficult.  - I would only include non-adherence as a management issue and not think that implies that the underlying disease process might be more treatment resistant.  - Non-adherences related to C2M.  - This is difficult to detect.  - It depends mostly on patient, so they should take care of that.  - Some non-adherent patients will have easy to treat, but hard to manage psoriatic disease.  - Common, could be related to nocebo effect.  - Non-adherence often relates to other conditions such as social and psychological factors, and partly often be compensated for by giving IV treatment or having a home nurse giving the patient the medicine.  - Is a question of management rather than of efficacy of a drug.  - If the patient is non-adherent (of course after adequate education by the treating physician) and this results in suboptimal disease control, then that is the patient’s problem and it should be explained as such to the patient: such a clinical situation does not deserve the term difficult-to-treat.  - Would consider this rather a management issue, nothing wrong with the treatments and switches are unlikely in this setting.  - Lack of adherence to the proposed therapeutic plans.  - The patients are untreated, having a severe disease and usually in need lot of care and support.  - Should be considered, but would not include it in the total number of drugs used unless adherent for at least a certain period, e.g., 6 months to a year, before discontinuing. OR if non-adherence is due to non-response.  - Most of the times non adherence is due to financial constraints and hence cannot be considered as D2T. it is totally a different reason where patients discontinue medication despite efficacy and tolerance.  - Very often patients not take their medication, missed the doses.  - This is potentially a correctable feature can be hard to establish definitively.  - Most of the time patients get better with MTX effective dose weekly about 20 mg / week. But the left once skin leasing has gone. Most of the patient they do not think back pain is due to psoriasis .. if skin lesion gone, they left the medication. Next time they might be developed drug resistance or compliance of the drugs, did not respond well.  - Should be caused by: individual reasons, accident, policy such as COVID-19 restrict.  - Drug intolerance or contraindication due to Side effect, related condition (uveitis, IBD).  - Drug intolerance could be patient factors that are difficult to quantify objectively (intolerance could be due to needle phobia, could be due to worsening "fatigue" which is hard to quantify if from drug or disease or something else, patient anxiety etc). Therefore, intolerance needs to only be in difficult to manage because it is not necessarily true drug failure or a difficult to treat PsA, the therapy may be stopped due to completely unrelated patient factors unrelated to true PsA  - Drug intolerance is common. In addition, after certain time pts do not like to continue injections and ask for oral meds instead.  - I think non-adherence should be addressed separately and include the patient point of view in the management.  - D2T should be reflective of biologically refractory disease despite optimal treatment, therefore drug adherence should not be included.  - Drug non-adherence might be managed with improved interaction with the prescribing doctor.  - Difficult to treat definition should not encompass subjects that cannot be grouped as defined by the response to treatment. Lack of adherence is a prototypical patient management point.  - Patients who do not adhere to treatment should be considered for referral to psychologists, psychiatry or thanatology, to become aware of the importance of pharmacological treatment and eliminate that obstacle in order to evaluate the response or not to treatment.  - Assuming this is not intolerance (difficult to tell apart sometimes) and is just non-adherence then I think this is a social/management issue more than medical.  - Verificar la adherencia debe ser previo al concepto de falla terapéutica o intolerância.  - Best to avoid muddying the water. I would keep the definition focused.  - From a research or management standpoint, I don't see any value in combining together patients who have tried multiple medicines without benefit on certain domains with those who haven't really tried. It would be like classifying a patient as difficult to treat/mange if they never showed up to clinic.  - It's the same idea of the last question.  - Failure to comply on pharmacy instructions like frequency / dosing / ....  - It is important BUT it is not failure of inflammatory control. I would include it in difficult to manage  - Assume or prove compliance.  - Adherence issues are not within uncontrolled problems.  - Same as above. I would include non-adherence as an exclusion for difficult to treat. If a patient is non-adherent to the medication that drug will not work, so it is not difficult to treat, that patient is difficult to manage.  - It is also a continuum.  - I feel that this is not applicable.  - It is a reason for difficult to manage.  - Non-adherence fits into difficult to manage.  - D2T should be restricted to true resistance to modes of action tried.  - Compliance is a huge problem that have an influence on the success of the treatment. |
| What additional clinical issues or comorbidities should be addressed in the workup and management of D2T and C2M-PsA | - Diabetes with neuropathy.  - Cardiovascular comorbidities, obesity.  - Liver tests.  - Cardiovascular disease, obesity, mental health, sleep, functional limitations.  - As mentioned, chronic regional soft tissue inflammation (bursitis in particular) has created considerable difficulty for a number of my PsA patients (C2M). Hepatosteatosis.  - Neuropsychiatric comorbidities, non-adherence or intolerance to medications, non-inflammatory pain, chronic fatigue.  - All extra musculoskeletal manifestations.  - I think the whole initiative of defining what is difficult to treat/ manage might be a wrong way to go, as there are so many "faces" of patients who can be considered difficult to treat/manage for many reasons. Maybe it would be clearer and more productive to define who can be considered as a RESPONDER (or an "easy to treat" patient) and state that everyone not fulfilling this definition is difficult to treat/manage?  - C2M: socio-economic status and personal beliefs may affect choice of therapy. Affordability may affect choice of the required advanced therapy or monitoring. I have come across religious beliefs impacting on choice of therapy (presence of animal protein). D2T: Previous malignancies are not categorised above, though I suspect it is in the exhaustive list. Has the time come now to be more open about the safety of bDMARDs in this area and open the option to most people? This may help in managing some D2T with more effective agents.  - Obesity, fibromyalgia, pain from pre-existing joint damage.  - Diabetes, hypertension, cardiovascular diseases, gout.  - As noted above, C2M-PsA patient reluctance to start appropriate therapy particularly in this era of shared decision making. May in part fall into the anxiety category, but truly a bit different.  - Fibromyalgia, depression.  - Axial PsA: Under-reported, under-recognized, heterogenous/often incomplete assessment, thereafter may not be addressed in clinic to sway treatment decisions. This might contribute to damage that are not prevented as it wasn't addressed "early", and may contribute to "chronic pain" due to damage/degeneration. Metabolic (CVD, DM, HT, Hlipid) comorbidities. Bone health/osteoporosis: More work needs to be done. Psychological health, its determinants, its place in being part of holistic care (it’s a small component at this point); I think it impacts the incidence of concomitant fibromyalgia and affects assessment by physicians/clinical decision-making.  - Obesity / Metabolic syndrome (including NAFLD) - Psychiatric comorbidities.  - BMI, compliance, health belief, pain sensitization or fibromyalgia, anxiety, depression.  - Obesity, liver disease including HBV, HCV, chronic kidney disease, previous or indolent infection i.e., tuberculosis, HZV, cytopenia.  - Severe obesity, with BMI >3 0 (due to its impact on aetiopathogenesis and TNFi response). Smoking. due to its impact on aetiopathogenesis and TNFi response). Concomitant IBD, as these patients behave differently to standard PsA patients in terms of symptoms, imaging signs and treatment response / options.  - MASE, DM, depression Hypertension.  - D2M, fibromyalgia, metabolic syndrome.  - Other pain, fibromyalgia or neuropathic pain, difficult to manage PsA.  - DM, Obesity should be recognised as pro inflammatory states which can potentiate psoriatic inflammation. Chronic pain, mood disorders should be considered for contribution to burden of pain and fatigue which may not necessarily be inflammation driven.  - Cardiovascular risk.  - The impact of PsA therapies on comorbidities.  - Patient skipping more than 2 doses of csDMARDs or bDMARDs consistently over 3 visits.  - Maybe can be considered as part of difficult-to-manage C2M PsA instead.  - IBD or possible IBD is one of the more challenging aspects in my experience. Comorbidities such as gout, obesity etc. can contribute to D2T PSA.  - Number of comorbidities.  - D2T - Co-morbidities (IBD, AFB, etc.) may affect drug choice. C2M - Adherence, weight control, drug access are all important issues.  - For D2T: metabolic comorbidities; for C2M: psychiatric disorders, fibromyalgia, and concomitant osteoarthritis.  - Central sensitization.  - Coexistence of fibromyalgia or osteoarthritis, coexistence of obesity (minor drugs response).  - PsA patients often have co-morbidities and other competing rheumatologic conditions such as OA, gout, central sensitization and lacks regular training, which are important to diagnose and handle.  - Every kind of cancer.  - Anything that influences/ restricts treatment options- like family history of demyelination, prior cancers.  - Depression, anxiety, cardiovascular diseases, obesity.  - Adherence, side effects.  - Depression, anxiety, obesity, hypertension, osteoporosis, diabetes etc.  - Non-adherence may exist due to different factors, managing factors; anxiety, forgetting, otherwise. Important to guide patients with non-adherence. Because otherwise we put aside a bDMARD, which isn't tried.  - Obesity, diabetes, liver function, inflammatory bowel disease.  - CV and malignancy risk.  - Non-adherence should be part of complex to manage.  - Any comorbidity you have to consider so the number of treatment options will be reduced.  - CVD, depression, osteoporosis, NAFLD.  - Obesity, immunocompromised with infection, multiorgan failure, etc.  - Presence of inflammatory eye and bowel disease, significant renal or liver impairment, inflammatory markers, BMI, damage joint count.  - Close monitoring and if two Biologics different action does not work need to consider tofacitinib combined with MTX.  - Complex to manage PsA include social issue (e.g., cost may contribute NOT to use bDMARDs/tsDMARDs).  - Non adherence is an issue more so in countries where health service support is poor. Also, pts do not like to continue injections.  - Non-adherence is purely a patient factor even though it could be due to intolerance. However, that should not be included in difficult to treat PsA as it is not technically a disease or therapy failure - therefore, the patient may not be a difficult to treat PsA but only other factors causing them to be non-adherent and therefore active disease or "fail" multiple therapies.  - Metabolic syndrome (obesity, diabetes, ...), as part of the C2M group.  - Mental health, any condition affecting physical function directly and indirectly.  - Active inflammatory disease and nociceptive pain can also occur in some patients so this group would fit both definitions. Ultrasound or MRI is often the only way to know what is going on. Nociceptive pain will include both central sensitisations as peripheral sensitisation  - - Cardiovascular disease because they may exclude JAKi.  - Fibromyalgia should be included in the difficult to treat and obesity in the difficult to manage.  - Anxiety/Depression, IBD, psoriasis, obesity  - IBD, Depression and Anxiety, arthrosis  - Lack of access, or even loss of access, to certain drugs (especially the newer ones, more expensive ones and in low-income countries) is crucial to define true lack of response or difficulty of management.  - Amount of domains involved. Obesity already mentioned but very important.  - Recurrent infections, fibromyalgia, depression and anxiety.  - Obesity, Liver Disease, Osteoporosis.  - Related conditions (if present) as has a big impact on treatment options. For D2M - also other comorbidities that may influence symptoms.  - Adult Rheumatologists are probably better able to address that question than am I (Pediatric Rheumatologist). Maybe consider fibromyalgia / centralized pain.  - Depression and anxiety. Intolerance and adherence to medication.  - Comorbidities like metabolic syndrome and NASH, previous infections and fibromyalgia.  - Medical diseases like diabetes, Hypertension and PMH like cancers - Smoking history - Pain and fatigue status - Open mind for pain related to osteoarthritis or gout - Secondary fibromyalgia / depression.  - PASI outcomes, IGA outcomes, and NAPSI outcomes.  - A time element.  - Liver fibrosis, metabolic syndrome.  - For complex to manage: all what was mentioned above, and any other conditions that prevent some drugs to be used. For difficult to treat the severity of activity should be included. A patient that is in remission in all domains, except mild skin, should probably not be defined as D2T.  - All known comorbidities.  - Objective side effects.  - Obesity, Depression.  - Obesity, depression, lack of education, inability to afford medications are additional factors.  - Obesity, Smoking, Osteoporosis, Family planning, History of malignancy.  - Diabetes, increased liver enzymes, skin disease, cardiac failure should be addressed in order to manage D2M  Comorbidities, smoking, adherence.  - I think that personality structure should be addressed as we know that it influences on compliance. |
| Please define any additional characteristics and suggest criteria for D2T-PsA | - Persistent objective evidence of inflammation in the context of being on b/tsDMARD should be required (not just symptoms). Elevated CRP or ESR should not be a compulsory element.  - Primary and secondary failure, side effects and contraindications of medications.  - I would not split these two concepts and find the survey not optimal in this regard (as it pushes me to do so)  - As csDMARD remains first choice in many countries, failure of such should be included in the criteria. Mode-of-action of the failed treatment might add valuable insights in different manifestations.  - Consider whether patient needs to have tried a therapy for a minimum amount of time before calling it a failure, e.g., 3 months.  - Comorbidities affecting the ability to use certain medications, e.g., heart failure and TNFi.  - Steroid use.  - Rapid secondary failures to advanced therapeutics.  - Rapid radiographic progression.  - Disease activity measures including MDA and PASDAS should be included in the defining D2T-PsA.  - In our study, we identified complete lack of response to methotrexate as being predictive of a disease profile which doesn't have sustained response to other therapeutic options.  - Chronic pain (fibromyalgia), quality of life.  - Enthesitis, nail disease, depression, chronic pain or nociplastic pain, obesity.  - Cancer, hypertension, diabetes, MACE.  - Separate primary from secondary loss of efficacy.  - Misdiagnosis.  - A specific category of D2T PsA could be those patients with true differential response on different PsA manifestations (e.g., excellent skin response on IL23i, but less optimal rheumatological response; differential efficacy for axial and peripheral involvement; issues with inflammatory bowel disease and/or uveitis): probably this is only a small subgroup, but in this very specific setting combination therapy should be explored.  - ERS-SA risk score calculator.  - Depression.  - Imaging confirmation of inflammation, would like this to be in no unclear terms.  - Low social class patients with poor understanding of the disease and/or difficult to obtain expensive biologic therapy.  - Patients with cancer and other chronic infections (HBV, latent TB, etc.)  - Genetic patient try most of biologic and still active and flare.  - Presence of inflammatory eye and bowel disease, significant renal or liver impairment, inflammatory markers CRP, ESR, BMI.  - Difficult to treat is a narrow category indicating unresponsiveness to drug. Consider renaming as Treatment-Resistant PsA or Drug-Resistant PsA.  - Not yet.  - Elderly pt. Community support. Risks of infections. Multiple comorbidities.  - Difficult to treat should be purely objective findings. Complex to manage should include contraindications to modalities (i.e., MS etc. too) in addition to comorbidities. That can make a PsA C2M.  - axSpA. Advanced disease in respect to joint destructions. Disease duration.  - - Differentiation between joint size. A swollen knee usually has a larger impact on a patient than a single swollen smaller joint  - Pain Vas >2 should be included  - Patients with rapid radiologic progression (less than 6 months)  - I am only a simple dermatologist Remaining stiffness and pain despite treatment. In Derm department we often encounter skin completely healed with remaining joint problems  - Clinical, imaging or biochemical evidence of persistent MSK or cutaneous inflammation.  - I think these were largely covered above. Difficult to treat would be active PsA (arthritis, dactylitis, enthesitis, psoriasis) despite treatments outlined above. For dactylitis, enthesitis, and sacroiliitis, would want imaging confirmation; this is not needed for peripheral arthritis.  - Composite scores with high value despite treatment.  - I would use this term for patients with treatment failure of 1 or more DMARDs and 2 or more b/tsDMARDs.  - Radiology positive finding.  - Patients with moderate to severe active disease in one or more of the PsA domains (Activity in that domain should be enough to drive a medication change by its own), in spite of at least one csDMARD and three b/tsDMARDs with different mechanism of action.  - Axial disease, Scalp disease.  - Starting point for both D2T and D2M is patient AND clinician impression of uncontrolled PsA (ie not just persistent unrelated acute phase or imaging in absence of symptoms - those relate to not achieving T2T which is not same as D2T/D2M). |
| Please define any additional characteristics and suggest criteria for C2M-PsA | - Regional chronic bursitis; fatty liver disease.  - I would not split these two concepts and find the survey not optimal in this regard (as it pushes me to do so)  - Non-adhesion, co-morbidities, e.g., obesity, osteoporosis, depression  - Lack of access to healthcare team/care facilities. This should include considerations for both primary & tertiary care facilities, including access to subspecialties whose help might be needed to manage the comorbidities.  - Obesity should not be included.  - Drug adherence, comorbidities, depression and chronic pain syndromes  - Fibromyalgia and Neuropathic pain, poor socioeconomic level.  - Osteoarthritis/degenerative disc disease and fibromyalgia should be a part of difficult to manage PsA.  - Concomitant drugs for central sensitization symptoms.  - Deleterious impact of PsA treatments on comorbidities (lung disease for methotrexate, IBD for IL17 blockers, etc.)  - Control of other comorbidities including diabetes, hyperuricemia and family stressors.  - In the real world, finances play a large role. If a patient does well with a biologic but cannot continue due to financial or insurance reasons, they then become difficult to manage so I would include that.  - Drug access through public payers or insurance.  - Cost, caregivers at home.  - In the opinion of the patient & provider, the disease is uncontrolled and the symptoms are intolerable despite multiple treatments.  - I am actually not very fond of this broad definition that is probably little or not helpful in daily practice (not to the treating physician, but in my view, there is also no benefit to the patient to be marked "complex to manage".  - Low literacy, low disease understanding.  - Low social class patients with poor understanding of the disease and/or difficult to obtain expensive biologic therapy.  - Non-adherence.  - Older age, female gender.  - Unresolved arthritis enteritis with multiple biologics.  - Patient drug tolerability listed, pain related to osteoarthritic joints or PsA damaged joints.  - Difficult to manage broadly includes anything that negatively impacts the patient's therapeutic journey or "closes doors" to certain treatment pathways.  - Should emphasize psychology and metabolic syndrome control and rehabilitation training.  - To evaluate whether early treatment can prevent D2T-PsA. To evaluate whether total disease burden correlates with D2T-PsA.  - Sex should be included Number of comorbidities (more than 2, for example or based on comorbidity indexes values).  - PtGA > 2 should be included.  - Patients with more than 1 metabolic comorbidity.  - Extra-cutaneous, extra-MSK disease manifestations (Uveitis; IBD), co-morbidities, drug intolerance, chronic pain, depression/anxiety, poor adherence.  - This could be more broadly defined and based in part upon symptoms even without active inflammation. I would also include drug intolerance or contraindications here (e.g., a history of infections precludes use of biologics). The only exception is csDMARDs -- inability to use/tolerate them should still be included in the difficult to treat rather than difficult to manage, the rational being that the effectiveness of csDMARDs in PsA is iffy, so a patient who has failed multiple biologics probably would not have responded great to methotrexate anyway.  - PRO with high pain/disability/impact of disease in QOL.  - I would use this term for patients with treatment failure or intolerance or contra-indication for 1 or more csDMARDs or 2 or more b/tsDMARDs, but we should evaluate each condition separately to define next steps.  - Fibromyalgia and other central sensitization syndromes  - Patients not fulfilling remission criteria or with poor quality of life, because of subjective measurements, without signs or symptoms of active disease, and patients with active disease because unable to receive proper treatment because of adverse events or comorbidities  - I cannot think of any.  - Should include gender, level of education and economic status.  - Mental health issues other than depression/anxiety. Pregnancy makes management difficult.  - Any intolerance to more than 2 drugs with different mechanisms, any of the diseases mentioned above should be mentioned in the definition of C2M.  - Starting point for both D2T and C2M is patient AND clinician impression of uncontrolled PsA (i.e., not just persistent unrelated acute phase or imaging in absence of symptoms - those relate to not achieving T2T which is not same as D2T/C2M) |

# **Supplementary Figures**

**Supplementary Figure S1.** Preferred imaging modalities.

**Supplementary Figure S2.** Factors contributing to difficult-to-treat psoriatic arthritis.

**Supplementary Figure S3.** Factors contributing to complex-to-manage psoriatic arthritis.
